# CD73 downregulation by EGFR-targeted liposomal CD73 siRNA potentiates antitumor effect of liposomal doxorubicin in 4T1 tumor-bearing mice

**DOI:** 10.1038/s41598-022-14392-7

**Published:** 2022-06-21

**Authors:** Anvar Soleimani, Farshad Mirzavi, Sara Nikoofal-Sahlabadi, Amin Reza Nikpoor, Bita Taghizadeh, Mehdi Barati, Mohammad Soukhtanloo, Mahmoud Reza Jaafari

**Affiliations:** 1 Department of Clinical Biochemistry, Faculty of Medicine, Mashhad University of Medical Sciences, Mashhad, Iran; 2 Department of Pharmaceutics, Faculty of Pharmacy, Hormozgan University of Medical Sciences, Bandar Abbas, Iran; 3 Molecular Medicine Research Center, Hormozgan Health Institute, Hormozgan University of Medical Sciences, Bandar Abbas, Iran; 4 Department of Medical Biotechnology, School of Advanced Medical Sciences, Tabriz University of Medical Sciences, Tabriz, Iran; 5 Nanotechnology Research Center, Pharmaceutical Technology Institute, Mashhad University of Medical Sciences, Mashhad, Iran; 6 Department of Pharmaceutical Nanotechnology, School of Pharmacy, Mashhad University of Medical Sciences, Mashhad, Iran

**Keywords:** Cancer, Drug discovery, Drug delivery

## Abstract

Blocking CD73 ectonucleotidase has been proposed as a potential therapeutic approach for cancer treatment. The present study aimed to investigate the antitumor effect of a novel EGFR-Targeted liposomal CD73 siRNA formulation in combination therapy with liposomal doxorubicin in the 4T1 mouse model. CD73 siRNA was encapsulated into nanoliposomes by the ethanol injection method. After preparation, characterization, morphology, and stability evaluation of formulations, the toxicity was measured by MTT assay. Uptake assay and efficiency of the liposomal formulations were investigated on the 4T1 cell line. The liposomal formulation containing CD73 siRNA was targeted with GE11 peptide for in vivo evaluations. Following biodistribution analysis, the antitumor activity of prepared formulations in combination with liposomal doxorubicin was studied in mice bearing 4T1 metastatic breast cancer cells. Finally, the induction of immune response of formulations in concomitant treatment with liposomal doxorubicin was evaluated in the tumor microenvironment of a mouse model of breast cancer. The size of prepared liposomal formulations at N/P = 16 for the liposomal CD73 siRNA and GE11-liposomal CD73 siRNA groups were 89 nm ± 4.4 and 95 nm ± 6.6, respectively. The nanoparticle’s PDI was less than 0.3 and their surface charge was below 10 mV. The results demonstrated that N/P = 16 yielded the best encapsulation efficiency which was 94% ± 3.3. AFM results showed that the liposomes were spherical in shape and were less than 100 nm in size. The results of the MTT assay showed significant toxicity of the liposomes containing CD73 siRNA during the 48-h cell culture. Real-time PCR and flow cytometry results showed that liposomes containing CD73 siRNA could effectively downregulate CD73 expression. Liposomal formulations were able to significantly downregulate CD73 gene expression, in vivo. However, CD73 downregulation efficiency was significantly higher for the targeted form compared to the non-targeted formulation (*P* value < 0.01). The combination showed maximum tumor growth delay with remarkable survival improvement compared to the control group. Studying the immune responses in the treatment groups which received doxorubicin, showed decreased number of lymphocytes in the tumor environment. However, this decrease was lower in the combination therapy group. Finally, our results clearly showed that CD73 downregulation increases the activity of CD8^+^ lymphocytes (IFN-ℽ production) and also significantly decreases the Foxp3 in the CD25^+^ lymphocytes compared to the control group. GE11-Lipo CD73 siRNA formulation can efficiently knockdown CD73 ectonucleotidase. Also, the efficacy of liposomal doxorubicin is significantly enhanced via the downregulation of CD73 ectonucleotidase.

## Introduction

Breast cancer is the most common cause of cancer death in women worldwide. It has been shown that Recurrence Rate for Triple-Negative Breast Cancer (TNBC) is high following surgery and conventional therapies^1^. Chemotherapy with anthracycline drugs including doxorubicin is the primary systemic chemotherapeutic approach for metastatic breast cancer. Following its administration, doxorubicin rapidly suppresses tumor development. Meanwhile, the efficiency of chemotherapy can be significantly reduced due to tumor resistance to anticancer drugs as well as unfavorable side effects^2^. Understanding the underlying molecular pathways in the microenvironment of advanced solid tumors including breast cancer, may help control cancer metastasis^3^. Solid tumor’s microenvironment (TME) comprises a variety of cell types including: tumor cells, stromal cells, and infiltrating immune cells. Alteration in the metabolic pathways of tumor cells as well as the cross-talk between cancer and immune cells in the TME, creates a TME-specific microenvironment with hypoxia, inflammation and low pH levels^4–7^. The consequences of these alterations favor tumor development via involvement of various molecular mechanisms^8^. Upregulation of CD73 is one of the major alterations in the tumor milieu of solid tumors^9,10^.

CD73, or 5'-Nucleotidase, is a glycoprotein enzyme attached to the extracellular surface of the cellular membrane through a glycosylphosphatidyl inositol (GPI) anchor^11^. CD73 is overexpressed in the hypoxic and inflammatory conditions of solid tumor’s TME and has been identified as a poor prognostic factor^12,13^. Furthermore, it has been recently observed that chemotherapy with doxorubicin also upregulates CD73 expression^14^. CD73 is involved in tumor progression via enzymatic and non-enzymatic mechanisms^15^. CD73 is a nucleotidase which converts AMP to adenosine and increases TME adenosine concentration to 1000–10,000 nM^16^. We have previously reported that CD73-derived adenosine has various effects on cancer cells, infiltrated immune cells and endothelial cells through binding to adenosine receptors (A1, A2A, A2B, and A3), which can lead to cancer progression. In particular, adenosine as an inflammatory mediator, strongly suppresses infiltrating immune cell’s activity, upon binding to its receptors^4^. Furthermore, it has been shown that CD73 may also participate in metastasis through interaction with the extracellular matrix^10,17,18^.

Identifying selective downregulation of genes by RNA interference (RNAi) mechanisms, has revolutionized molecular biology. Small interfering RNAs (siRNAs) are short double-stranded RNAs (19-bp) with 2 nucleotide overhangs on their 3′ ends^19,20^. The ability of siRNA to selectively knock down any target gene through RNA-induced silencing complex (RISC) has paved the way not only for research but also for the treatment of a variety of incurable diseases such as cancer. Despite its potential for selective downregulation of any target gene, siRNAs delivery to target cells is considered a great challenge in their in vivo studies^21^. Efficient gene silencing through the RNAi mechanism, requires an optimal delivery system. During recent years, many carriers including viral vectors, polymers, and nanolipoparticles have been developed for efficient delivery of RNAi molecules in vivo^22^. Efficient therapeutic RNAi delivery carriers should be non-toxic, avoid RNase activity, exhibit high transfection efficiency, and promote cellular uptake and endosomal escape. While viral vectors showed high transfection efficiency, they are considered as unsafe gene delivery vehicles for gene therapy due to their inflammatory and oncogenic potential. Lipid nanoparticles (LNPs) are recognized as one of the simplest and safest non-viral carriers for delivery of siRNA molecules^23,24^. Liposomes are considered the most suitable lipid nanoparticles for drug delivery due to various advantages including biocompatibility, biodegradability, high loading efficiency, and reduced toxicity^25^. Moreover, liposomes smaller than 100 nm in size can easily penetrate into tumor microenvironment due to enhanced permeability and retention (EPR) effect^26,27^. Considering high expression of epidermal growth factor receptor (EGFR) on the surface of advanced solid tumors, active targeting of liposomes with EGFR ligand (GE11), may increase the concertation of liposomal formulation in the TME. Over the past 10 years, various nanolipoparticles have been investigated as efficient delivery systems for siRNA in different cancers^19^. Recently and for the first time, Onpattro (Patisiran), a lipid nanoparticle-encapsulated siRNA, has been approved by the FDA for treatment of hereditary transthyretin-mediated amyloidosis (hATTR)^28^. Onpattro has opened new doors towards developing novel gene therapy approaches for treatment of incurable diseases via optimal nanoparticles for efficient knock down of any target gene.

Considering the key functions of CD73 molecule during tumor progression, several CD73 antibodies are now in clinical trials for treatment of solid tumors^29,30^. Since CD73 enhances cancer development via its enzymatic and non-enzymatic activities, its downregulation by a well-designed siRNA delivery system may provide a novel therapeutic strategy in cancer treatment. Therefore, the purpose of present study was to investigate the combinatorial treatment of EGFR-targeted liposomes containing CD73 siRNA and liposomal doxorubicin for treatment of metastatic breast cancer in mice.

## Material and methods

### Materials

1, 2-Distearoylsn-glycero-3-phosphoethanolamine-N-[methoxy (polyethylene glycol)-2000] (mPEG2000-DSPE), 1, 2-dioleoyl-sn-glycerol 3 phosphoethanolamine (DOPE) and 1, 2-dioleoyl-3-trimethylammonium-propane (DOTAP) were purchased from Avanti Polar Lipids (Alabaster, AL, USA), Octyl glucoside (OG), cholesterol and RPMI 1640 were purchased from Sigma-Aldrich. The lipofectamine 2000 was purchased from Invitrogen (USA). PE-conjugated anti-mouse CD73 (Cat No: 127205) and isotype-matched control monoclonal antibodies (mAbs) were purchased from Biolegend (USA). Phorbol 12-myristate 13-acetate (PMA)/ionomycin cocktail, Anti-mouse CD3-APC, anti-CD8a-PE-cy5, anti CD4-PE-cy5, CD25-FITC antibodies, anti-IFN-γ-FITC and antiIL-4-PE antibodies were purchased from BD Biosciences (San Diego, USA). CD73 siRNA and non-targeting siRNA (Cat No: D-001210-01-20) were obtained from Dharmacon (Thermo Fisher, Hilden, Germany). GE11 peptide (CGGGYHWYGYTPQNVI) with 1857 Da molecular weight and purity of 92.17% was purchased from China Peptides Co. (Shanghai, China). Liposomal Doxorubicin was purchased from Exir Nano Sina Company (Tehran, Iran). DEPC-treated water was applied in all experiments for siRNA containing samples. All other reagents were chemical grade.

### Preparation of nanolipoparticles

Nanolipoparticles containing CD73 siRNA were prepared by ethanol injection method^31^. Briefly, lipid compositions (DOTAP; DOPE; mPEG2000-DSPE; cholesterol) (Total lipid: 10.08 mM) were dissolved in chloroform at a molar ratio of 39.68; 39.68; 0.79; 19.84 respectively. To eliminate chloroform from the lipids and lipidic film preparation, rotary evaporation (2 h) and freeze-drying (2 h) were performed. In order to prepare 1 ml nanolipoparticles containing CD73 siRNA, 300 μl warm absolute ethanol (55 °C) was added to the lipidic film and dissolved by vortex mixing. The lipidic mixture was added to 700 μl of warm aquatic phase (45 °C) in a dropwise manner, while the aquatic phase was simultaneously vortex. The composition of aquatic phase of CD73 siRNA nanolipoparticles was chosen based on the charge ratio (N/P). For N/P = 4, N/P = 12, and N/P = 16, the aquatic phase was 10% sucrose solution containing 0.307, 0.102 and 0.076 mg of CD73 siRNA, respectively. For empty liposomes, the aquatic phase only contained sucrose/HEPES buffer (sucrose: 9.5%, HEPES 10 mM, pH7.4). The mixture of aquatic and lipidic phases were vigorously vortexed for 5 min and then sonicated for 45 s at 45 °C under argon gas exposure. After an overnight incubation at 4 °C, the nanoliposome formulations were passed through three polycarbonate filters with three pore sizes (200 nm, 100 nm, and 50 nm). Each formulation was passed 11 times through each filter. After that, ethanol dilution was performed by adding 2 ml sucrose/HEPES buffer to the formulations. Finally, in order to eliminate ethanol and non-encapsulated siRNA molecules, formulations were dialyzed against sucrose/HEPES buffer (sucrose: 9.5%, HEPES 10 mM, pH7.4) for 48 h at 4 °C using 100 kDa MWCO dialysis membranes. In order to prepare GE11-targeted liposomes containing CD73 siRNA, 100 µg/mL of GE11-maleimide-PEG2000-DSPE micelles were post-inserted into the liposomes containing CD73 siRNA at 55 °C and shaken for 4 h under argon gas exposure. The final liposomal formulation was sterilized by filtration through a 0.22 µm microbial syringe filter.

### Liposome characterization

#### Particle size, PDI, zeta potential, and encapsulation efficiency measurement

The mean hydrodynamic particle diameters, zeta-potential (ζ) and polydispersity index (PDI) of the empty liposomes and CD73 siRNA-loaded nanoliposomes were measured using dynamic light scattering (DLS) (Zeta Sizer Nano-ZS; Malvern Instruments Ltd., Worcestershire, UK). To measure the particle size and PDI, 70 μl of liposome formulations were diluted with 930 μl of sucrose/HEPES buffer. For zeta potential measurement, 20 μl of liposome formulations were mixed with 980 μl normal saline buffer (1 mM HEPES, pH: 7.4). The encapsulation efficiency of siRNA-loaded nanolipoparticles was measured in triplicate by UV spectrophotometry (SPEKOL 1300, Analytic Jena, Germany). In order to release encapsulated siRNA molecules, the liposomes were lysed by addition of 950 μl of octyl glucoside (OG) (200 mM) to 50 μl of the formulation. Following 5 min of vigorous vortex and water bath incubation at 37 °C for 30 min, the absorbance was measured at 260 nm using a UV spectrophotometer. The encapsulation efficiency was assessed using the following formula:

$${\text{Encapsulation}}\;{\text{Efficiency}}\;\left( \% \right) = {\text{CD}}73\;{\text{siRNA}}\;{\text{concentration}}\;{\text{after}}\;{\text{dialysis}}/{\text{Initial}}\;{\text{CD}}73\;{\text{siRNA}}\;{\text{concentration}} \times 100.$$

### Conjugation of GE11 peptide with maleimide-PEG 2000-DSPE

GE11 peptide-lipid conjugation involved peptide’s thiol groups at the C terminal of cysteine residues and maleimide’s pyrrole groups. Briefly, after dissolving GE11 in DMSO and maleimide-PEG2000-DSPE in chloroform, conjugation was performed at the molar ratio of 1.2:1 (peptide: maleimide) at 37 °C for 48 h. the conjugated mixture was lyophilized by using a rotary evaporator and a freeze dryer. The lyophilized powder was then hydrated with DEPC and GE11-maleimide-PEG2000-DSPE was dispersed by sonication in 25 °C water bath for 5 min. The conjugation of GE11-maleimide-PEG2000-DSPE was confirmed via thin-layer chromatography (TLC) and Tricin SDS-PAGE. After that, post-insertion method was used to prepare liposomal formulation with GE11 Peptide at the concentration of 50 μg/ml at 65 °C for 1 h. 120 GE11-maleimide-PEG2000-DSPE molecules were approximately quantified on the surface of each liposome. The following parameters were applied to calculate the number of GE11 peptide molecules per liposomes: (a) liposome average size: 100 nm in diameter; (b) phospholipids concentration of liposomal formulation: 8.07 mM; (c) phospholipid molecules per liposome with the average size of 100 nm: 8 × 10^4^; (d) the number of liposomes per each ml: 1 × 1014; (e) number of peptide molecules per each ml aliquots of peptide-micelles: 12 × 10^15^. Following post-insertion, the purification of the liposomal formulation containing GE11 peptide was carried out by dialysis against sucrose 5% buffer (pH 6.5) in dialysis cassettes (Spectrum) with 14 kDa molecular weight cut off (MWCO). The final liposomal formulations were sterilized by filtration through a 0.22 μm microbial syringe filter^26,32^.

### Stability studies

The physicochemical stability of liposomes containing CD73 siRNA was evaluated for 4-weeks storage period at 4 °C in the refrigerator. Changes in the particle size, PDI, and zeta potential were evaluated by DLS every week. The serum stability of CD73 siRNA-containing nanolipoparticles was evaluated in PBS/FBS (50/50, v/v) for 96 h at 37 °C. To evaluate the stability of our formulation, nanoliposomes containing CD73 siRNA were subjected to dialysis in a 100 kDa MWCO dialysis cassette against 100 ml PBS/FCS. At several time intervals (0, 2, 4, 8, 12, 24, 48, 72, and 96 h), samples were drawn from the dialysis bag and the percentage of remaining encapsulated CD73 siRNA were determined using the above-mentioned protocol. Furthermore, liposomes were also examined visually for signs of sedimentation and changes in their color during the tests period^33,34^.

### Assessing the surface morphology of liposomes by atomic force microscopy

The surface morphology of empty and CD73 siRNA-loaded liposomes (N/P = 16) were evaluated using atomic force microscopy (AFM). This method evaluates and provides three-dimensional images from the surface of nanoparticles including liposomes. Briefly, formulations were diluted with HEPES buffer (1: 500 for both empty and CD73 siRNA-loaded liposomes). In order to prepare AFM slides, 30 μl of each formulation was placed on mica slips and dried at room temperature. Finally, the prepared samples were observed at different magnifications and the images were analyzed by AFM microscope (NanoWizard^®^II, JPK model, Germany). During the cantilever tip approach to sample’s surface, imaging in 2, 5 and 10 μm scales were performed; therefore, 2D and 3D images of liposomes were obtained.

### Cell line

The 4T1 cell line was obtained from the National Cell Bank of Iran (Pasteur Institute of Iran, Tehran, Iran), and cultured in RPMI 1640 medium, supplemented with 10% FBS, penicillin (100 IU/ml) and streptomycin (100 IU/ml) and incubated in a humidified incubator containing CO2 (5%) at 37 °C.

### Cytotoxicity assay

The cytotoxicity of CD73 siRNA-encapsulated liposomes and free CD73 siRNA molecules were evaluated on 4T1 cell line by MTT assay. Briefly, 4 × 10^4^ cells/well were seeded in 96 well plates with complete medium and incubated overnight at 37 °C. After 24 h of incubation, cells were treated with CD73 siRNA-loaded liposomes (25 nM siRNA, 50 nM siRNA, 100 nM siRNA), Naked CD73 siRNA (25 nM and 100 nM), liposomes containing non-targeted siRNA (25 nM) and empty liposome (without siRNA) at 70–80% confluency in OPTI-MEM1 medium (Invitrogen^™^, USA) without FBS and antibiotics. The negative control group contained non-treated cells. Transfection was carried out during 6 h incubation in OPTI-MEM1 medium, without FBS and antibiotics. Then, 100 µl of FBS and antibiotics were added to the wells and incubated for 2 h and 48 h. After that, 5 mg/ml of MTT solution was added to each well and incubated for another 4 h in the dark at 37 °C. In order to dissolve the formazan crystals, 200 μl of dimethyl sulfoxide (DMSO) was added to each well and incubated at 37 °C for 15 min. Finally, the absorbance was measured at 570 nm by a microplate reader. All experiments were carried out in triplicate and the viability of non-treated control cells was considered 100%.

### Cellular uptake assay

To evaluate the uptake efficiency of siRNA-containing formulations in 4T1 cell line, CD73 siRNA labeled-Cy3 fluorescent dye was used and detected by both inverted fluorescent microscopy and flow cytometry methods. 4T1 cells (1 × 10^6^ cell/well) were seeded in 6 well plates in RPMI 1640 medium. After overnight incubation, cells were treated with nanoliposomes containing 25 nM CY3-labeled siRNA, 25 nM CY3-labeled siRNA/lipofectamine complex (positive control), 25 nM Naked CD73 siRNA and empty liposomes (negative control) in serum and antibiotic-free medium and incubated for 6 h in 37 °C. Next, cells were washed three times with PBS and were observed under an inverted fluorescence microscope. For flow cytometry analysis, cells were washed three times with PBS, followed by resuspension of each well in 500 μl of the staining buffer. Finally, FlowJo software was used to evaluate the uptake efficiency.

### In vitro analysis of CD73 gene downregulation

To evaluate the knockdown efficiency of liposomes containing CD73 siRNA, 3 × 10^4^ of 4T1 cells were seeded into 24 well plates in RPMI 1640 medium (10% FBS) without antibiotics, at 37 °C for 24 h in a humidified incubator containing 5% CO2. After an overnight incubation, cells were divided into five groups including liposomal CD73 siRNA (25 nM), CD73 siRNA/lipofectamine complex (25 nM) (positive control), naked CD73 siRNA (25 nM), and empty liposome (negative control). Cells were transfected with the optimized concentration of siRNA (25 nM) in FBS and antibiotic-free OPTI-MEM medium at 40 to 60% confluency for 6 h. After this time, the medium was replaced with complete OPTI-MEM (10% FBS, 100 units/ml penicillin and100 μg/ml streptomycin) and cells were incubated at 37 °C for another 48 h. After this final incubation time, cells were trypsynised and collected for evaluating the levels of CD73 mRNA and protein.

#### RNA extraction and quantitative real-time reverse transcription PCR (qRT-PCR)

Total RNA was extracted using RNA isolation kit (Qiagen, Germany) and cDNA was synthesized from extracted total RNA using QuantiTect Reverse Transcription kit (Qiagen) according to the protocol. RT-qPCR was carried out using a SYBR Green master mix (amplicon), cDNA template (50 ng, 2 μl), and primers (0.5 μl) in the final volume of 12 µl in a light cycler (Roche) real-time PCR system. The primer sequence for CD73 gene and GAPDH were: CD73 forward primer: 5′-TCCTGCAAGTGGGTGGAAT C-3′, CD73 reverse primer: 5′-TAGATGGGCACTCGACACTTG-3′; GAPDH forward primer: AATGGATTTGGACGCATTGGT, GAPDH reverse primer: TTTGCACTGGTACGTGTTGAT. The thermocycler parameters were set according to the master mix (amplicon) protocol as follows: all samples were pre-incubated at 95 °C for 5 min and amplified 35 cycles at 95 °C for 15 s and 58 °C for 30 s (2 step amplification). All samples were run in duplicate. Melting curve analysis was performed to evaluate product quality and the rate of non-specific amplification. GAPDH was selected as the housekeeping gene and internal control. Relative CD73 gene expression was normalized against GAPDH and analyzed by the ΔΔCT method.

#### Flow cytometry analysis

To evaluate gene silencing by nanoliposomes containing CD73 siRNA, the expression of CD73 ectonucleotidase was evaluated by flow cytometry. After treatment time, 4T1 cells of each group including Liposomal CD73 siRNA (25 nM), siRNA/lipofectamine complex (25 nM) (positive control), naked CD73 siRNA (25 nM), and empty liposome (negative control) were harvested and washed twice with the washing buffer (PBS containing 0.5% BSA and 0.1% NaN_3_). Then, cells were stained with PE rat anti-mouse CD73 and PE Rat IgG2b isotype control mAb in 50 µl PBS washing buffer at 4 °C for 30 min in the dark. After an additional three times wash, cells were suspended in 500 µl washing buffer and analyzed by flow cytometry (BD FACS Calibur^™^, BD Biosciences, San Jose, USA).

### In vivo studies

#### Animal and ethics statement

Female BALB/c mice (4–6 weeks) were purchased from Royan Institute (Tehran, Iran) and kept under the appropriate conditions. All animal studies were carried out according to the rules of the Institutional Ethical Committee and Research Advisory Committee of Mashhad University of Medical Sciences. This study was approved by the Institutional Ethical Committee and Research Advisory Committee of Mashhad University of Medical Sciences (Ethic No. IR.MUMS.fm.REC.1396.11) and complied with the ARRIVE guidelines (https://arriveguidelines.org).

#### Biodistribution study

Tumor induction was performed by injection of 4 × 10^5^ 4T1 tumor cells into the right flank of each mouse subcutaneously. After nine days of tumor inoculation, mice with palpable tumors were randomly categorized into four groups (three mice in each group). Animals in each group were intravenously injected with GE11-liposomes and non-targeted liposomes containing Cy3-CD73 siRNA, naked Cy3-siRNA, and PBS. Animals were sacrificed at 4, 24, and 48 h post injection and their organs including heart, lung, kidney, liver, spleen and tumor tissue were removed and washed in normal saline. The Cy3 fluorescent dye was detected in the tumor tissue and other organs using fluorescence images obtained by animal imaging technique. Finally, imageJ/FIJI tool was used to quantify the fluorescent intensity in different tissues of treated groups at 4, 24, and 48 h post injection.

#### Anti-tumor study

The experiment was performed on 70 female BALB/c mice, which were previously inoculated with 4T1 cells. For tumor inoculation, mice were first anesthetized via intraperitoneal injection of ketamine-xylazine (K, 100 mg/kg; X, 10 mg/kg). In order to evaluate the anti-tumor effectiveness of prepared formulation in combination with liposomal doxorubicin, 4 × 105 4T1 cells were injected into each mouse and after nine days, mice with palpable tumors were divided into seven different groups (10 mice in each group) including PBS, naked CD73 siRNA (5 µg/mice), liposomal negative control siRNA (NC siRNA) (5 µg/mice), liposomal CD73 siRNA (5 µg/mice), GE11-liposomal CD73 siRNA (5 µg/mice), combination (liposomal doxorubicin (5 mg/kg) + GE11-liposomal CD73 siRNA (5 µg/mice)), and liposomal doxorubicin (5 mg/kg). The formulations containing CD73siRNA were injected intravenously twice per week and for four weeks, while a single dose of liposomal doxorubicin was injected into relevant groups. Bodyweight and tumor volume was measured during the time of the study. Time-to-event endpoints (TTE), median survival time (MST), tumor growth delay (%TGD), and increase in lifespan (%ILS) were also determined for each experimental group. From each group, five mice were sacrificed after the last injection at the fourth week and tumor tissues were removed to evaluate CD73 expression, tumor weight, and immune responses. The remaining mice (five mice in each group) were followed up to evaluate the survival rate.

#### Tumor CD73 expression and CD73 level in tumor tissue

In order to evaluate the effect of formulations containing CD73 siRNA on downregulation of CD73, in the fourth week of the experiment, three mice from each group were sacrificed, tumors were isolated and RNA was immediately extracted from the tumor tissues using RNA extraction kit. Then, cDNA was synthesized and real-time PCR was performed according to the protocol mentioned in the in vitro study section. Moreover, CD73 levels in tumor cell suspensions was measured by staining with anti-mouse CD73 antibody. Considering that CD73 is a peripheral ectonucleotidase and is located on the surface of tumor cells, the process of staining was carried out the same as tumor-infiltrated lymphocytes staining approach.

#### Isolation of tumor-infiltrated lymphocytes

In order to isolate tumor-infiltrated lymphocytes (TILs), three mice in each group were sacrificed through injection of 100 µl of ketamine-xylazine solution. Tumors were immediately removed and washed twice with cold PBS, under sterile condition, and cut into tiny pieces with a sterile scalpel in a warm (37 °C) collagenase type I solution (2 mg/ml in PBS). In the next step, the digest was quenched by adding RPMI-1640 medium supplemented with 10% FBS. Subsequently, cells were harvested with the cell strainer and washed excessively with cold PBS. In addition, erythrocytes were lysed with ACK lysing buffer (0.15 M NH4Cl, 1.0 M KHCO3, 0.1 mM Na2EDTA). The remaining cells were re-suspended in RPMI-1640 medium supplemented with 10% FBS.

#### Flow cytometry analysis of isolated cells

Flow cytometry (FCM) analysis of TILs was performed using the FACS Caliber flow cytometer (BD FACS Caliber^™^, BD Biosciences, San Jose, USA). Approximately 1.0 × 10^5^ cells were seeded and cultured in a 24 well plate containing 1 ml of RPMI-1640 10% FBS per well. Afterward, cells were stimulated with 2 ml/ml of PMA/ionomycin plus Brefeldin-A cocktail (BioLegend, San Diego, CA) for 6 h at 37 °C. Then, cells were harvested by centrifugation at 250 × g for 5 min, washed twice with PBS (containing FBS 2%), and stained according to the supplier’s instructions (Cytofix/Cytoperm TM Plus Fixation/Permeabilization, BD Biosciences, California, USA). Briefly, the cell suspension was stained with cell surface marker antibodies (2 ml/tube), i.e., mouse anti CD73, mouse anti-CD8a-PE-cy5, mouse anti-CD3-APC, mouse anti-CD25-FITC, and mouse anti-CD4-PE-cy5 antibodies, for 20 min in the dark at 4 °C. Cellular suspensions were then washed with PBS (containing FBS 2%) using centrifugation (250 g for 5 min). Then, they were fixed and permeabilized with Cytofix/Cytoperm buffer solution (BD Bioscience) for 20 min. Next, cells were stained with fluorescent-labeled specific antibodies against intracellular cytokine (2 ml/tube) in Perm/Wash buffer (BD Bioscience) for 30 min at 4 °C. Antibodies included mouse anti-IFN-g-FITC, mouse anti-IL-4-PE, and mouse anti-Foxp3-PE. Cells were washed with Perm/Wash buffer and re-suspended in PBS (containing 2% FBS). Finally, the frequency of cells producing specific surface and intracellular markers were determined using BD FACS Caliber (BD Bioscience) flow cytometry.

### Statistical analysis

Statistical analysis was carried out using Graph Pad Prism version 6 (Graph Pad Software, USA). Independent samples t-test and post-hoc test for one-way ANOVA were used. Tukey's multiple comparisons were used to evaluate the differences among groups. A *P* value < 0.05 was considered statistically significant. Data are presented as Mean ± SD. Survival studies were analyzed by log-rank test in Graph Pad Prism software.

## Results

### Characterization of liposomal formulations

Physicochemical characteristics of the formulations including size, zeta potential, PDI, and CD73 siRNA encapsulation efficiency are shown in Table 1. The average particle size was different for nanoliposome containing CD73 siRNA with different N/P ratios. We observed that the N/P ratio significantly affects nanoliposome’s size and encapsulation efficiency (EE). While the mean size of all nanoparticles was less than 150 nm in all groups, the optimum particle size was observed in N/P = 16 (89 nm). As presented in Table 1, nanoparticles had smaller sizes prior to dialysis (elimination of ethanol and unloaded siRNAs) in all liposomal formulations. The surface charge (zeta potential) of nanoliposomes was near to neutral. The best encapsulation efficiency (EE) in siRNA-containing formulations was obtained in N/P = 16.

**Table 1:** Effect of N/P ratio on size, zeta potential, and encapsulation efficiency (EE) of nanolipoparticles containing CD73 siRNA.

Formulation (preparation method)	N/P ratio	Particle size (nm) ± SD	PDI	Zeta potential (mV) ± SD	Encapsulation efficiency Percentage ± SD
**Ethanol injection**		Before dialysis			
Liposomal CD73 siRNA (DOTAP: DOPE: Cholesterol: mPEGDSPE (10.08 mM))	4	218 ± 13	0.21 ± 0.04	4 ± 0.5	97.2 ± 2.7
		After dialysis			
		221 ± 11	0.31 ± 0.05	9.8 ± 4.2	33.1 ± 5.2
**Ethanol injection**		Before dialysis:			
Liposomal CD73 siRNA (DOTAP: DOPE: Cholesterol: mPEGDSPE (10.08 mM))	12	186.4 ± 12.6	0.12 ± 0.03	8 ± 2	96.7 ± 3.2
		After dialysis:			
		191.3 ± 8.2	0.10 ± 0.02	8.1 ± 2.5	66 ± 4
**Ethanol injection**		Before dialysis			
Liposomal CD73 siRNA (DOTAP: DOPE: Cholesterol: mPEGDSPE (10.08 mM))	16	85 ± 9	0.171 ± 0.04	4 ± 0.8	97.8 ± 1.2
		After dialysis			
		89 ± 4	0.30 ± 0.05	6 ± 2	94 ± 3
**Ethanol injection**		Before dialysis			
EGFR-Targeted Liposomal CD73 siRNA (DOTAP: DOPE: Cholesterol: mPEGDSPE (10.08 mM))	16	92 ± 5	0.181 ± 0.05	5 ± 1.5	97.1 ± 2.4
		After dialysis			
		95 ± 6	0.22 ± 0.07	7 ± 2.3	94 ± 3
**Lipidic film**		After dialysis:			
Empty liposome ((DOTAP: DOPE: Cholesterol) (10 Mm)	–	132.9 ± 21.8	0.242 ± 0.02	18 ± 4	–
		After dialysis			
		–	–	–	–

### Physicochemical stability of liposomes containing CD73 siRNA

**Figure 1:**
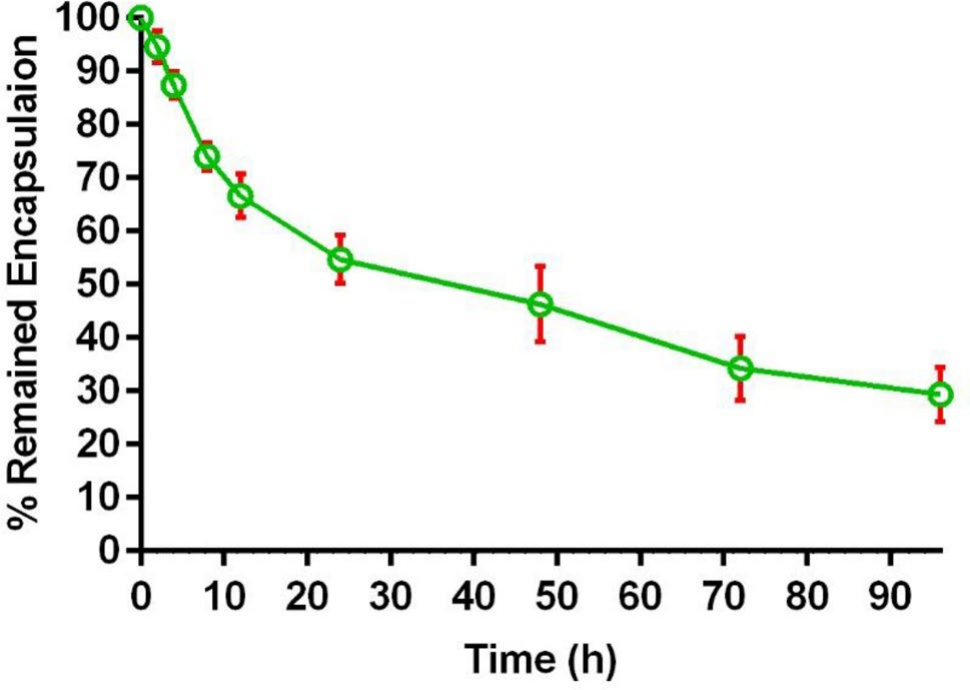
Liposomal formulation containing CD73 siRNA were incubated in the PBS/FBS medium at 37 °C for 96 h. The amount of remained siRNA reached near to 50% after 24 h incubation in the PBS/FBS medium at 37 °C and it decreased to around 30% after 96 h incubation. Data are shown as mean ± standard deviation (n = 3).

Physicochemical stability of liposomes containing CD73 siRNA was preserved without any significant alteration in their size, PDI, and zeta potential during 4-week incubation in the refrigerator (Table 2). The stability studies of siRNA-loaded nanoliposomes maintained in PBS/FBS medium showed that 50% of the siRNA molecules remained encapsulated after 24 h of incubation. Finally, CD73 siRNA content of the nanoliposomes decreased to 30% of their primary content after 96 h of incubation in a physiologically simulated medium (Fig. 1). Furthermore, we did not observe any liposomal CD73 siRNA aggregation or sedimentation during performing serum stability or preserving liposomes in the refrigerator.

**Table 2:** Monitoring the size, PDI, and zeta potential of Liposomal CD73 siRNA (DOTAP: DOPE: Cholesterol: mPEGDSPE (10.08 mM)) during 4 weeks storage period in the refrigerator.

Time (week)	Z-average (nm)	PDI	Z-potential (mV)
0	85 ± 4	0.17 ± 0.04	6 ± 2
1	87 ± 6	0.18 ± 0.06	6.5 ± 1.9
2	89 ± 5	0.19 ± 0.02	6.1 ± 2.1
3	90 ± 7	0.19 ± 0.05	6.2 ± 2.4
4	91 ± 8	0.18 ± 0.04	6.3 ± 2.7

All data are presented as mean ± SD of three independent experiments.

*PDI* Poly Dispersity Index.

### Assessment of liposome’s morphology by atomic force microscopy

**Figure 2:**
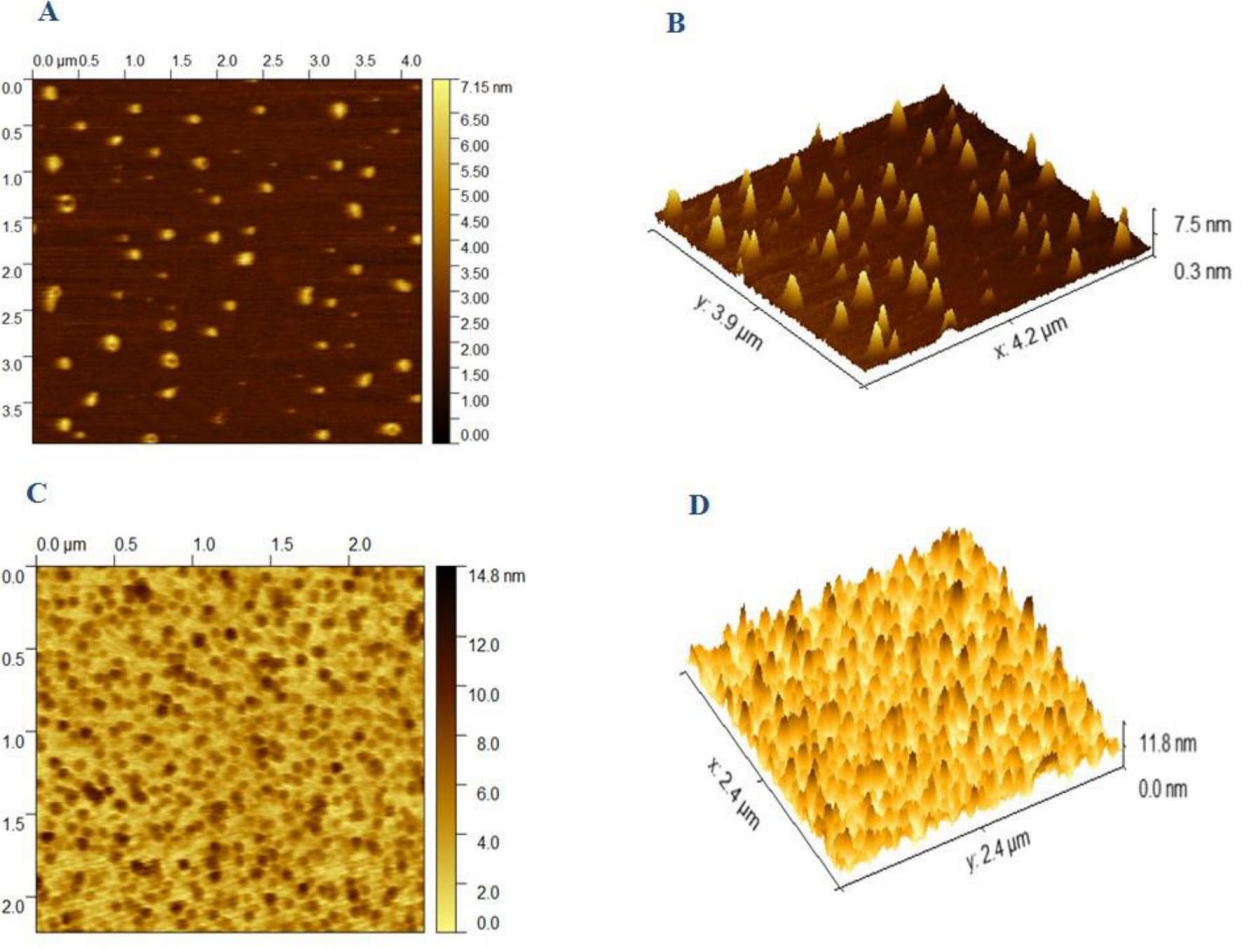
Morphology of empty liposomes (**A**, **B**) and liposomes Containing CD73 siRNA (**C**, **D**) by atomic force microscopy (AFM). Both empty liposomes and liposomes containing CD73 siRNA have spherical shape with high resolution and an average size of less than 100 nm. Figure (**A**) and (**C**) show the two-dimensional (2D) image of empty liposome and liposomes containing CD73 siRNA respectively. Figure (**B**) and (**D**) show the Three-dimensional (3D) images of empty liposome and liposomes containing CD73 siRNA respectively.

Figure 2 presents the morphology of empty and CD73 siRNA-loaded liposomes. Morphology analysis by AFM showed that both empty liposomes and liposomes containing CD73 siRNA had homogenous shape and size less than 100 nm which were in correlation with sizes obtained from DLS analysis.

### Thin layer chromatography (TLC) and SDS-PAGE results

To evaluate that the GE11 peptide coupling reaction to the mPEG2000-DSPE phospholipid was completed, TLC and SDS-PAGE tests have been carried out. Results showed that linking between GE11 peptide and phospholipid is performed. The GE1-DSPE lipid linkage stayed on the top of the SDS-PAGE gel due to its higher molecular weight and the completion of the reaction can be assured that no stain of product is aligned with the free GE11 peptide (Supplementary Fig. 1A and 1B).

### Cellular uptake of CD73 siRNA-loaded liposomes

**Figure 3:**
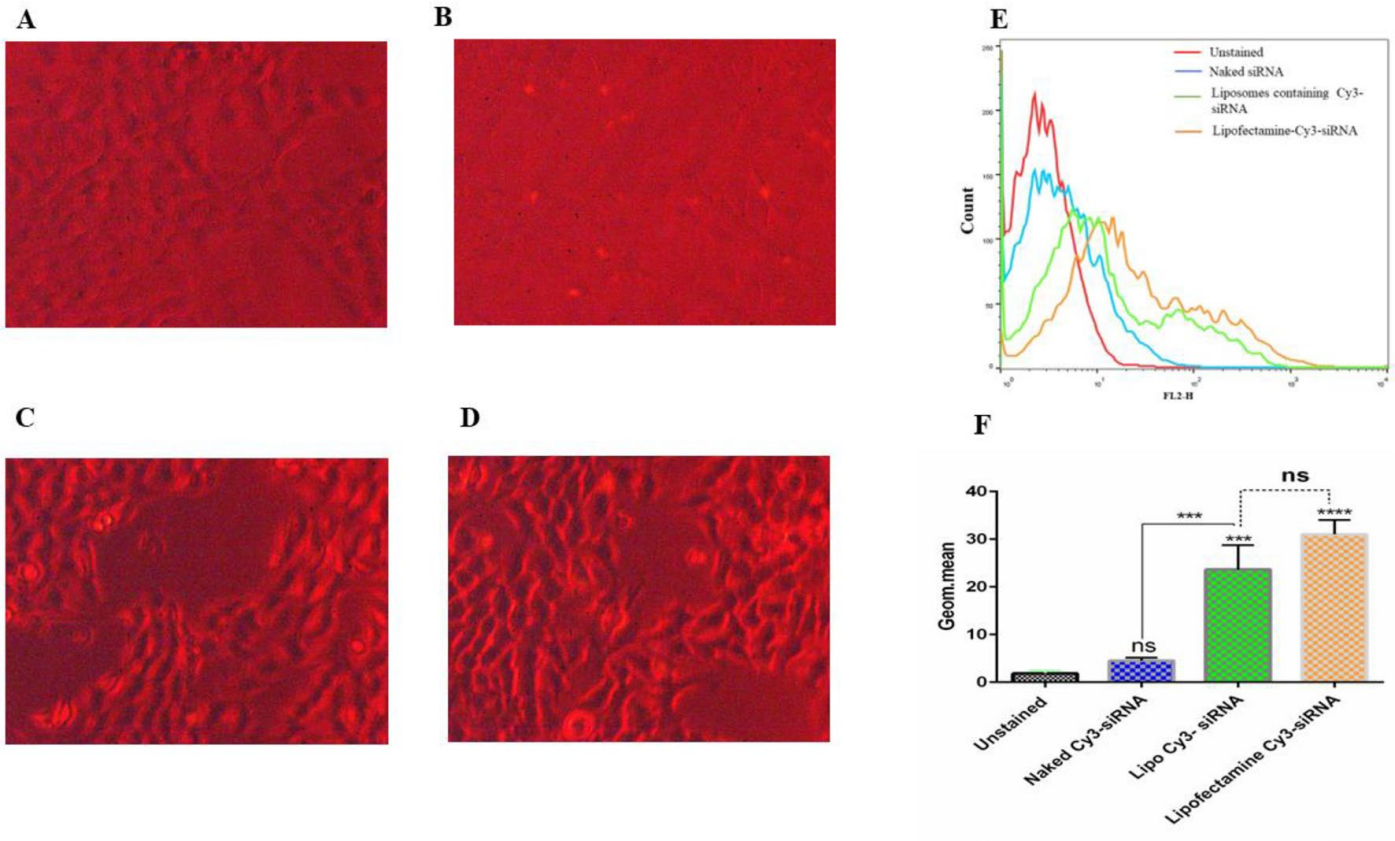
Uptake assay of liposomes containing Cy3-siRNA and Lipofectamine Cy3-CD73 siRNA in the 4T1 cancer cells. (**A**) The untreated 4T1 cells. (**B**) Un-liposomal Cy3-siRNA (Naked Cy3-CD73 siRNA). (**C**) Liposomes containing Cy3-siRNA. (**D**) lipofectamine Cy3-siRNA transfected cells. (**E**) The histogram analysis of siRNA uptake in 4T1 cells with liposome and lipofectamine compared to Naked siRNA. (**F**) Geometric mean (geom. mean) analysis of Cy3-siRNA uptake in 4T1 cells with liposome and lipofectamine. Data are shown as mean ± standard deviation (n = 3). Statistically significant differences are represented as follows: ***P < 0.001, ****P < 0.0001. The images are taken at 40X magnification.

The uptake efficiency of different treatment groups including liposomes containing Cy3-siRNA, Cy3-siRNA/lipofectamine complex (positive control), and naked Cy3-siRNA was evaluated in 4T1 cell line using fluorescent microscopy and flow cytometry analysis. The fluorescence microscopy results showed that 4T1 cells could effectively uptake liposomes containing siRNA, which is comparable to lipofectamine (positive control) (Fig. 3). The results of naked Cy3-labeled siRNA showed that it couldn’t be up taken by 4T1 cells. Analysis of flow cytometry results also demonstrated that 4T1 cells could uptake liposomes containing Cy3-siRNA (Fig. 3E). Fluorescence geometric means of 4T1 cells incubated with liposomes containing Cy3-CD73 siRNA is significantly higher than the naked form (*P* value < 0.001) and negative control (*P* value < 0.0001) (Fig. 3F). Meanwhile, the comparison of uptake assay between liposomes containing Cy3-siRNA and lipofectamine Cy3-siRNA had not any significant differences (*P* value > 0.05) (Fig. 3F).

### Cytotoxicity assay

**Figure 4:**
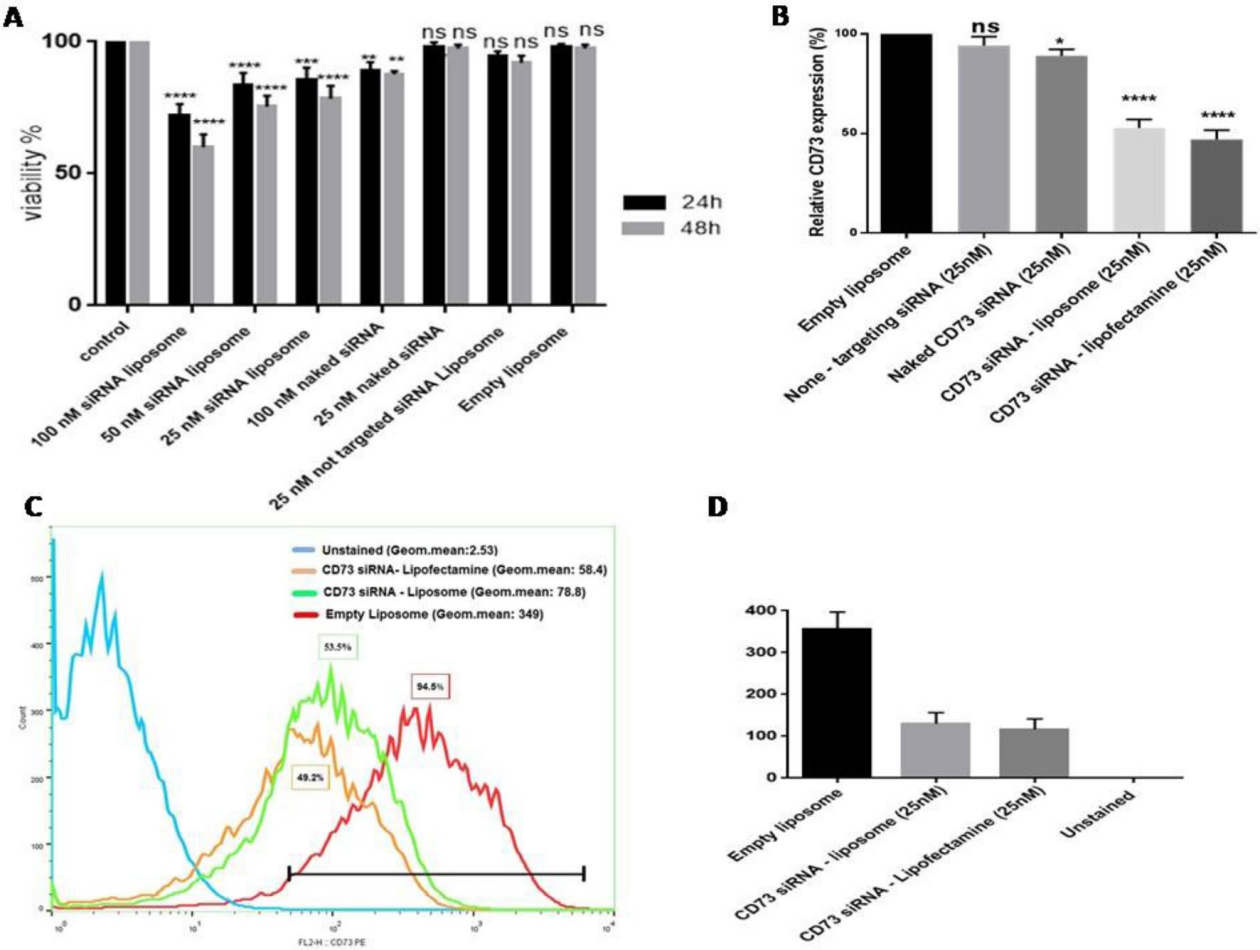
Evaluation of siRNA-mediated downregulation efficiency and toxicity of CD73 siRNA in 4T1 cell line. (**A**) Cytotoxicity of formulations including liposomes containing CD73 siRNA (100, 50, and 25 nM), naked CD73 siRNA (25 and 100 nM), non-targeting siRNA (25 nM), and empty liposomes was evaluated by MTT assay. The data are the mean ± SD of triplicate experiments. (**B**) After 48-h incubation of 4T1 cell with different formulations, the impact of liposomal CD73 siRNA and lipofectamine-CD73 siRNA (positive control) on the level of CD73 mRNA were assessed by real time PCR method. Both liposomes containing CD73 siRNA (25 nM) and lipofectamine CD73 siRNA complex (25 nM) can inhibit CD73 mRNA expression level up to 51%, but the naked form of CD73 siRNA and non-targeting siRNA could not affect CD73 expression effectively. (**C**, **D**) The results of flow cytometry analysis also demonstrated that liposomes containing CD73 siRNA and lipofectamine transfected group decrease the CD73 expression as compared with the empty liposome group.

The cytotoxicity of different formulations including liposomes containing CD73 siRNA (25, 50, and 100 nM CD73 siRNA), liposomes containing non-targeting siRNA (25 nM), Naked CD73 siRNA (100 nM, 25 nM), and empty liposomes was studied by MTT assay. Our results demonstrated that while liposomes containing CD73 siRNA (25, 50 and 100 nM CD73 siRNA) and Naked CD73 siRNA (100 nM) have significant toxicity on 4T1 cells, 25 nM Naked CD73 siRNA, liposomes containing non-targeting siRNA (25 nM), and empty liposomes did not show any significant toxicity on these cells during 24 h and 48 h treatment experiments (Fig. 4A).

### CD73 gene expression analysis

We evaluated the mRNA level of CD73 in 4T1 cells by qRT-PCR analysis. As illustrated in Fig. 4B, liposomes containing CD73 siRNA significantly decrease the level of CD73 mRNA expression compared to empty liposomes (negative control). The ability of our liposomes containing CD73 siRNA in downregulating CD73 gene was near to the lipofectamine-CD73 siRNA (positive control) (47.3% for siRNA-loaded liposomes and 52.7% for lipofectamine-CD73 siRNA treated group). While the naked form of CD73 siRNA was able to knock down the CD73 gene (10.9%), its efficiency was not comparable to liposomes containing CD73 siRNA. The results of liposomes containing non-targeting siRNA also showed no significant CD73 downregulation in comparison with untreated cells (negative control) (Fig. 4B).

### CD73 protein expression analysis

To demonstrate the efficiency of nanoliposomes containing siRNA on CD73 protein expression in comparison to lipofectamine-CD73 siRNA (positive control) and empty liposomes (negative control), the protein expression of CD73 was determined in 4T1 cells. As shown in Fig. 4C, both lipofectamine-CD73 siRNA (53.5%) and siRNA-loaded nanoliposomes (49.2%) substantially downregulated CD73 protein expression compared to empty liposomes (negative control). Furthermore, the mean fluorescence intensity (MFI) for lipofectamine-CD73 siRNA, nanoliposomes containing CD73 siRNA, empty liposomes and unstained groups were 58.4, 78.8, 349 and 2.53, respectively, which shows a significant decrease in MFI of lipofectamine-CD73 siRNA (58.4) and nanoliposomes containing CD73 siRNA (78.8) compared to empty liposome group (Fig. 4D).

### In vivo results

#### Biodistribution of targeted and non-targeted liposomal formulations containing Cy3-siRNA

**Figure 5:**
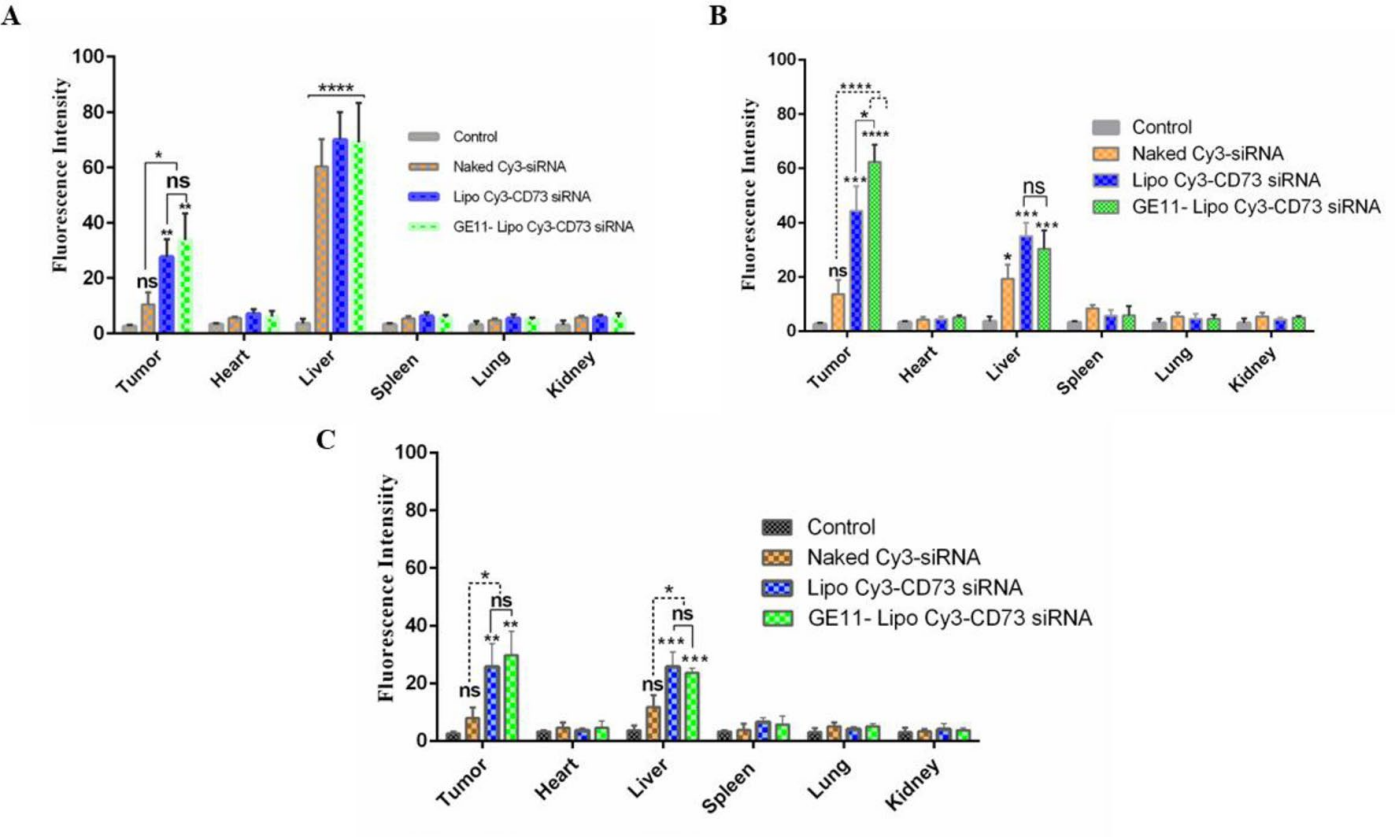
Tissue biodistribution of GE11-targeted, non-targeted liposomal formulation containing Cy3-siRNA, and Naked Cy3 siRNA at three-time intervals (4, 24 and 48 h post injection) in tumor site, heart, liver, spleen, lung, and kidney tissues. (**A**) Tissue biodistribution of different formulations at 4 h time interval. (**B**) Tissue biodistribution of different formulations at 244 h time interval. (**C**) Tissue biodistribution of different formulations at 48 h time interval. The intensity of Cy3 fluorescence dye indicates drug accumulation in different tissues. Data are expressed as mean ± standard deviation (n = 3). Statistically significant differences are represented as follows: *P < 0.05, **P < 0.01, ***P < 0.001, ****P < 0.0001.

siRNA labeled with Cy3 florescent dye was used to detect the presence of liposomal formulations in different tissues. As shown in Fig. 5, targeted liposomes showed the higher fluorescence intensity in tumor tissue. The analysis of fluorescence intensity showed that both targeted and non-targeted liposomes containing Cy3-siRNA had significant differences compared to Naked Cy3-siRNA at 4 h post injection (*P* value < 0.05) (Fig. 5A). Liposomal Cy3-siRNA and targeted liposomal Cy3 siRNA showed highest fluorescence intensity at the tumor site at 24–48 h following injection of formulations. Moreover, statistical analysis of fluorescence intensity showed that targeted liposomal Cy3 siRNA has significant differences compared to the non-targeted liposomal formulation (*P* value < 0.05) (Fig. 5B). the results of fluorescence intensity following 48 h post injection showed that the intensity of Cy3 siRNA is reduced in tumor tissue and other organs including liver. However, the fluorescence intensity of liposomal formulations (both targeted and non-targeted) was higher compared to Naked Cy3 siRNA and negative control (PBS). Liver tissue showed the highest Cy3 florescence intensity in both GE11-targeted and non-targeted liposomes at 24–48 h post injection. Although naked Cy3-siRNA was not significantly observed in the tumor tissue at three different time intervals of 4, 24 and 48 h, it was significantly observed in the liver tissue at different time intervals.

#### In vivo therapeutic efficacy in 4T1 tumor model of mice

**Figure 6:**
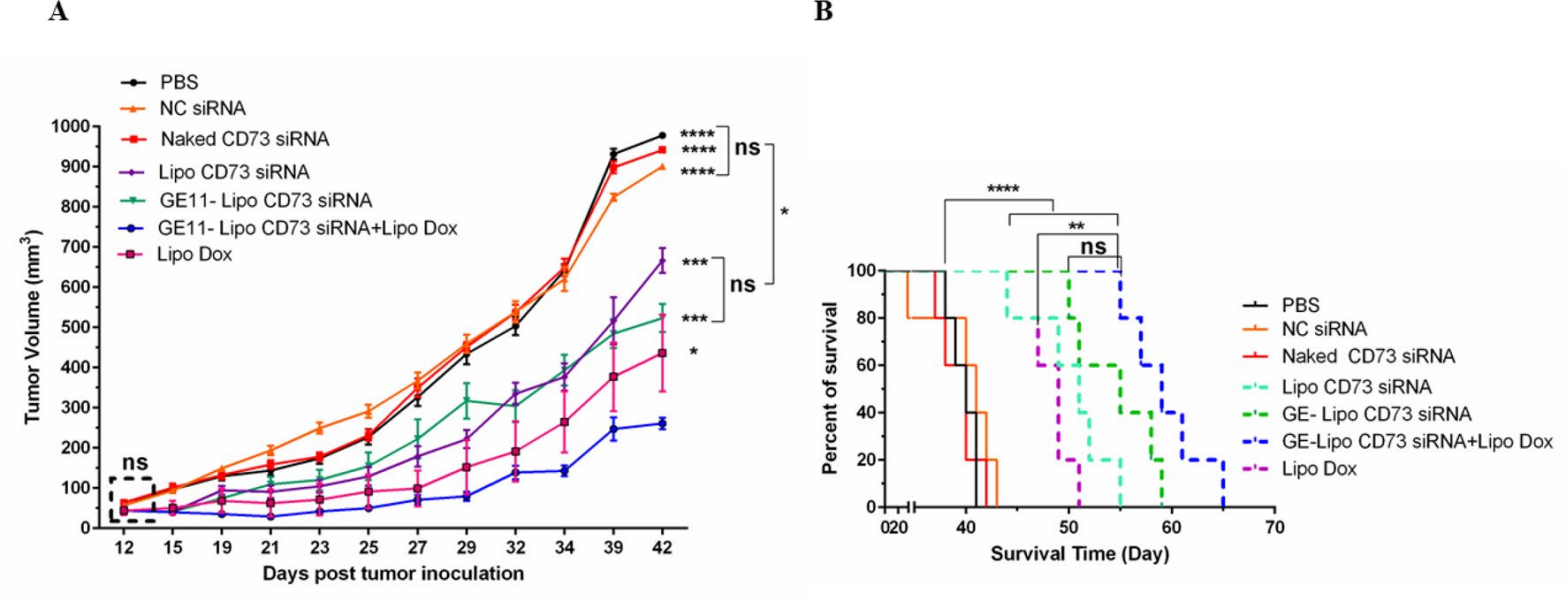
Therapeutic efficacy of liposomal CD73 siRNA, GE11-liposomal CD73 siRNA, liposomal doxorubicin, and GE11-liposomal CD73 siRNA+ liposomal doxorubicin combination in female BALB/c mice bearing 4T1 tumor. (**A**) Mean tumor volume in all groups. (**B**) Survival analysis of therapeutic groups (The log-rank test was used to analyze the survival). Data are expressed as mean ± standard deviation (n = 5). Statistically significant differences are represented as follows: *P < 0.05, **P < 0.01, ***P < 0.001, ****P < 0.0001.

**Figure 7:**
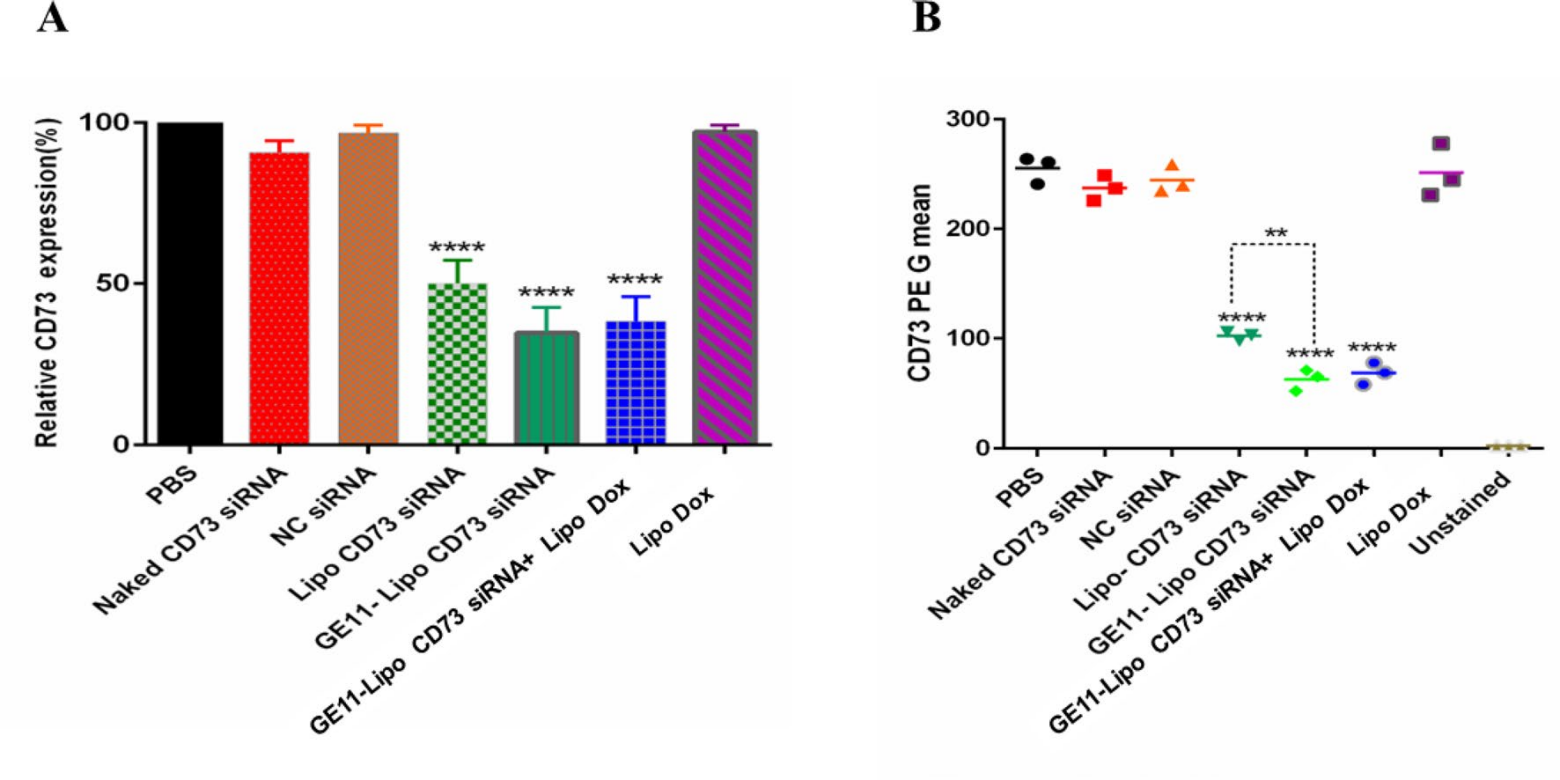
Efficacy of liposomal formulations on both CD73 gene expression and CD73 ectonucleotidase level by RT-PCR and flow cytometry, respectively, in the tumor tissue of mice inoculated with 4T1 cell line. (**A**) The result of RT-PCR showed that the targeted form of liposome containing CD73 siRNA (GE11-Lipo CD73 siRNA) alone and in combination with Liposomal doxorubicin could efficiently reduce the expression of CD73 gene (****: *P* value < 0.0001). (**B**) Decreased fluorescence intensity indicates decreased CD73 ectonucleotidase level. Histogram shows the intensity of PE fluorescence in the treatment groups. CD73 PE G mean stands for geometric mean of fluorescence intensity of CD73 PE. Data are shown as the mean ± SD (n = 3). Statistically significant differences are shown as follow: **P < 0.01 and ****P < 0.0001.

In order to determine the therapeutic efficacy of targeted and non-targeted liposomes containing CD73 siRNA in combination therapy with liposomal doxorubicin (Lipo Dox), several parameters including tumor growth and survival rate were monitored during 70 days post tumor inoculation. The results of tumor volume and survival rate are shown in Fig. 6. The comparison of tumor volume at 42 days post-tumor inoculation showed that combination group (GE11-Lipo CD73 siRNA+ Lipo Dox) has significant differences compared to PBS, Naked siRNA, Liposomal CD73 siRNA, GE11 liposomal siRNA, and Liposomal doxorubicin groups (*P* < 0.0001, *P* < 0.0001, *P* < 0.0001, *P* < 0.001, *P* < 0.001, *P* < 0.05 respectively). Combination therapy with GE11-Lipo CD73 siRNA+ Lipo Dox could more efficiently suppress the rate of tumor growth compared to liposomal doxorubicin and GE11-Lipo CD73 siRNA alone (*P* < 0.05 and *P* < 0.001, respectively) (Fig. 7A). Log-Rank analysis showed that substantial alterations in the survival rate of treatment groups compared to control groups (*P* value < 0.0001). Detailed analysis of tumor growth and survival rate showed that the combination group (GE11-Lipo CD73 siRNA+ Lipo Dox) could efficiently increase the survival rate compared to Liposomal doxorubicin group (*P* < 0.001) (Fig. 7B). MST and ILS (%) are considered the most important survival rate parameters. evaluation of these two parameters showed that the combination therapy with GE11-Lipo CD73 siRNA+ Lipo Dox, demonstrated higher MST (59) and ILS (47.5%) compared to Lipo Dox and GE11-Lipo CD73 siRNA alone (Table 3).

**Table 3:** Therapeutic efficiency of different liposomal formulations in 4T1 tumor mice model (n = 5).

Groups	TTE^a^ (days ± S.D.)	TGD^b^ (%)	MST^c^ (day)	ILS^d^ (%)
PBS	40.72 ± 1.15	–	40	–
Naked CD73 siRNA	41.57 ± 1.57	2.08	40	0
Negative control siRNA (NC siRNA)	41.8 ± 2.93	2.66	41	2.5
Lipo CD73 siRNA	50 ± 3.58	22.8	51	27.5
GE11-Lipo CD73 siRNA	53.4 ± 2.94	31.5	55	37.5
GE11-Lipo CD73 siRNA+ Lipo Dox	57.8 ± 7.86	41.96	59	47.5
Lipo Dox	46.75 ± 1.79	22.5	49	22.5

^a^Time to reach end-point.

^b^Tumor growth delay.

^c^Median survival time.

^d^Increase in life span.

#### Quantitative reverse transcription-PCR (qRT-PCR) in 4T1 mice model

As shown in Fig. 7A, targeted liposomal formulations containing CD73 siRNA (GE11-Lipo CD73 siRNA) alone and in combination with liposomal doxorubicin were able to significantly reduce the expression of CD73 gene to 61.8% ± 7.6 and 65.3%. ± 7.8, respectively, compared to the control group (*P* value < 0.0001). The liposomal form of CD73 siRNA was able to significantly downregulate CD73 expression up to 49.1% (*P* value < 0.0001). Naked CD73 siRNA and negative control siRNA (NC siRNA) were not able to significantly reduce CD73 siRNA expression (*P* value > 0.05).

#### Analysis of CD73 downregulation in 4T1 mice model by flow cytometry

As shown in Fig. 7B, CD73 siRNA-loaded and GE11-targeted liposomal formulations significantly downregulate CD73 levels compared to the control group (*P* value < 0.0001). However, comparison of liposomal CD73 siRNA (Lipo CD7 siRNA) with GE11-Lipo CD73 siRNA showed that the targeted form decreased CD73 levels more efficiently (*P* value < 0.01).

#### Flow cytometry analysis of tumor-infiltrated lymphocytes (TILs)

##### Analysis of tumor-infiltrated lymphocytes

**Figure 8:**
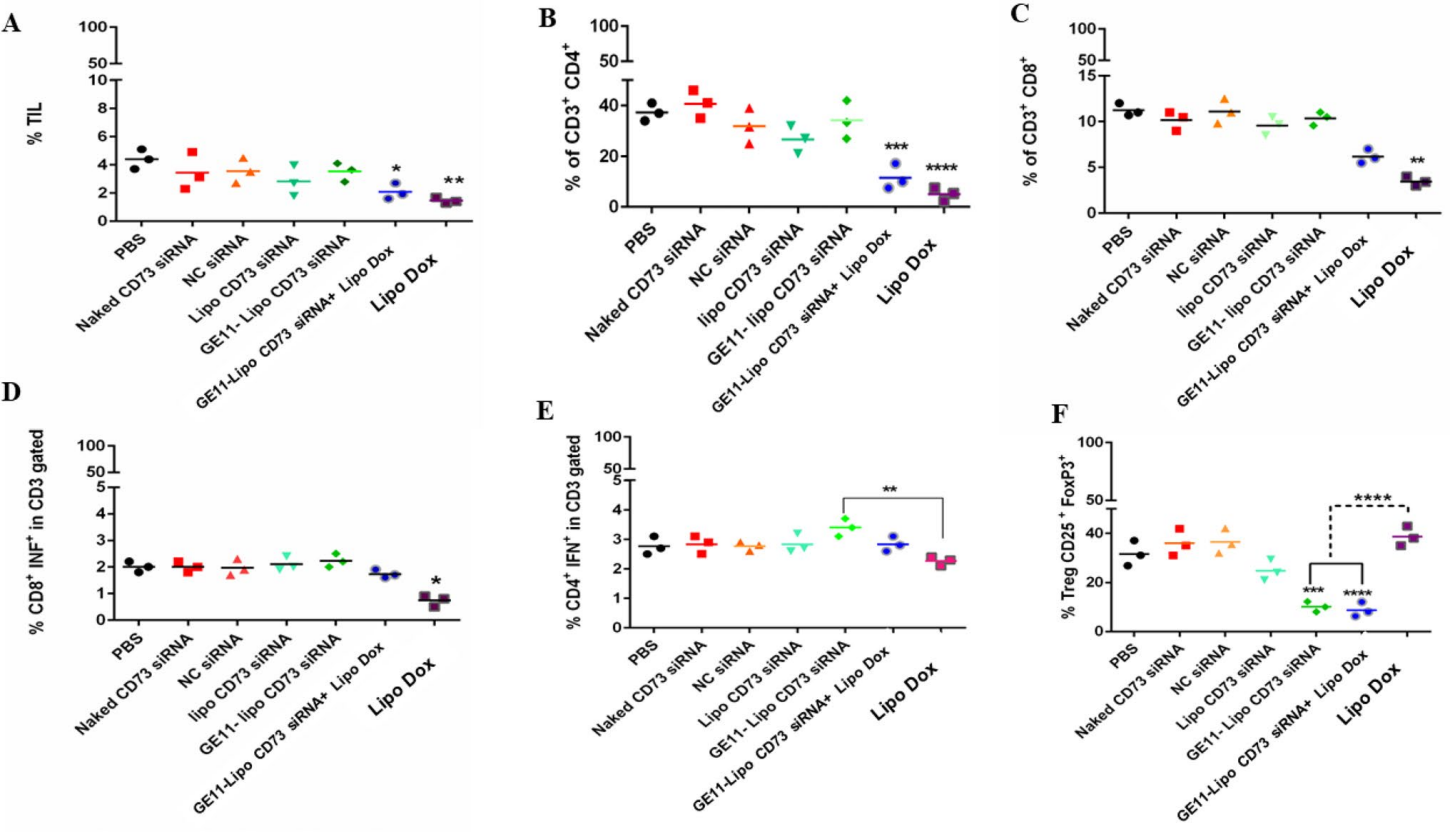
Frequency of tumor-infiltrated lymphocytes (TILs) and T cell subpopulations in the tumor environment of different groups. Two days after the last injection at the fourth week, three mice from each treatment group were sacrificed and cells from tumor tissue were isolated. Isolated cells were stained with conjugated antibodies against surface markers (CD3, CD4, CD8, and CD25 FoxP3). FACS analysis was carried out to determine the frequency of lymphocytes secreted into the tumor site. (**A**) The frequency of TIL following injection of mice with different formulations. (**B**) Liposomal formulation alone or in combination with GE11-Lipo CD73 siRNA decreased the frequency of CD3/CD4 positive lymphocytes. (**C**) Lipo Dox formulation group significantly decreased the frequency of CD3/CD8 positive lymphocytes (*P* value < 0.01). (**D**) Lipo Dox formulation negatively affected the frequency of CD8^+^, INF^+^ lymphocytes. In combination group, the negative effect of Lipo Dox formulation is attenuated by GE11-Lipo CD73 siRNA formulation. (**E**) Frequency of CD4^+^, INF^+^ in CD3 gated lymphocytes. (**F**) Effect of different formulation on the frequency of Treg CD25^+^, Foxp3^+^ lymphocytes. Data are reported as mean ± SD (n = 3). Statistically significant differences are represented as follows: *P < 0.05, **P < 0.01, ***P < 0.001, ****P < 0.0001.

Tumor-infiltrated lymphocytes (TILs) analysis showed that the frequency of TIL in the tumor environment of Liposomal doxorubicin, GE11-Lipo CD73 siRNA+ Liposomal doxorubicin, GE11 liposomal siRNA, liposomal siRNA, negative control siRNA (NC siRNA), Naked CD73 siRNA, and PBS-treated groups are equal to 1.46 ± 0.12, 2.07 ± 0.32, 3.51 ± 0.38, 2.82 ± 0.62, 3.56 ± 0.51, 3.46 ± 0.76 and 4.32 ± 0.39, respectively. Treatment of mice with Doxorubicin significantly reduced the number of TIL in both groups including liposomal doxorubicin (*P* < 0.05) and GE11-Lipo CD73 siRNA+ liposomal doxorubicin (*P* < 0.01) (Fig. 8A). The results of TIL subpopulations analysis are presented in Fig. 8. Chemotherapy with doxorubicin negatively and significantly decreased the frequency of CD4^+^ and CD8^+^ T lymphocytes in mice treated with liposomal doxorubicin and combination therapy (GE11-Lipo CD73 siRNA+ liposomal doxorubicin) groups. Meanwhile, GE11-Lipo CD73 siRNA in combination with liposomal doxorubicin could also attenuate the cytotoxic effects of Doxorubicin. Figure 8B,C clearly show that while liposomal doxorubicin significantly decreased the frequency of CD8^+^, treatment with GE11-Lipo CD73 siRNA could not decrease the frequency of CD8^+^ T cells at tumor site. The result of Fig. 8D showed that the frequency of CD8^+^ IFN^+^ in the liposomal doxorubicin group has significant differences from the control group (*P* < 0.05). On the other hand, the analysis of CD4^+^ INF^+^ in CD3 gated cells showed that there is not any significant difference between the liposomal doxorubicin group and the control group. However, the comparison of liposomal doxorubicin with GE-Lipo CD73 siRNA showed significant differences (Fig. 8E). Moreover, treatment of mice with GE11-Lipo CD73 siRNA alone and in combination with liposomal doxorubicin could significantly decrease the frequency of CD25^+^ FoxP3^+^ Treg cells compared to control group. Interestingly, these treatment groups showed significant differences with mice treated with liposomal doxorubicin alone (*P* < 0.0001) (Fig. 8F).

##### Analysis of intracellular cytokines in T cells subpopulations

**Figure 9:**
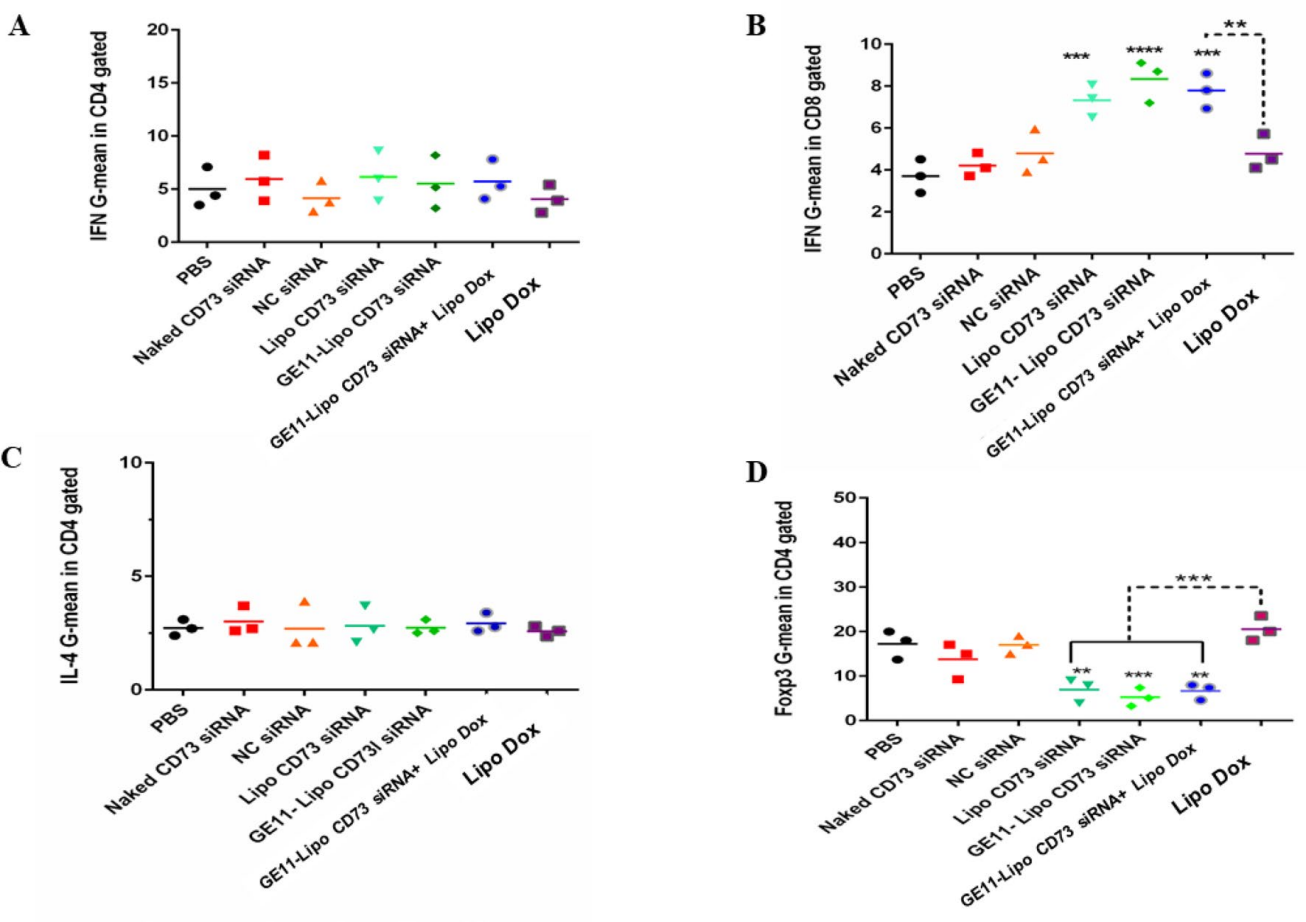
Intracellular cytokine profile of tumor-infiltrated lymphocytes (TILs). Two days after the last injection at the fourth week, three mice from each treatment group were sacrificed and tumor tissue cells were isolated. Isolated cells were stained with intracellular markers. Fluorescence intensity (geometric mean) of cytokines including IL-4, IFN-γ, and FoxP3 were assessed by flow cytometry. (**A**, **C**) Different formulations had not any significant differences on the geometric mean of IFN and IL-4 in the CD4^+^ gated cells. (**B**) Injection of Liposomal siRNA, GE11targeted siRNA-loaded liposomes and the combination group (GE11-Lipo CD73 siRNA+ Lipo Dox) markedly increased the geometric mean of IFN-γ in CD8^+^ T cells. (**D**) On the other hand, these treatments significantly decreased the florescent intensity of FoxP3 compared to control (PBS) and Liposomal doxorubicin groups. Data are reported as mean ± SD (n = 3). Statistically significant differences are represented as follows: *P < 0.05, **P < 0.01, ***P < 0.001, ****P < 0.0001.

Analysis of intracellular cytokines secreted by CD4^+^ (IFN-γ and IL-4) and CD8^+^ (IFN-γ) T cells were carried out by flow cytometry and results are presented in Fig. 9. Different liposomal formulations showed no significant effect on IFN-γ fluorescence intensity in CD4^+^ cells compared to control (*P* value > 0.05). Meanwhile, liposomes containing CD73 siRNA, GE11 Lipo CD73 siRNA and combination therapy (GE11 Lipo CD73 siRNA+ Lipo Dox) could significantly increase the geometric means of IFN-γ in CD8^+^ T cells (Fig. 9B). As shown in Fig. 9C, no significant differences were observed in the florescence intensity of IL-4 in CD4^+^ T cells following treatment with different liposomal formulations compared to control group (PBS) (*P* > 0.05). Analysis of FoxP3 florescence intensity in CD4^+^ T cells showed that liposomes containing CD73 siRNA, GE11 liposomal siRNA, and the combination group could efficiently decrease FoxP3 florescence intensity in comparison to the control group. Furthermore, comparison of Liposomal doxorubicin group with combinatorial treatment (GE11 Lipo CD73 siRNA+ Lipo Dox) demonstrated significant differences (*P* < 001) (Fig. 9D).

## Discussion

In the present study, liposomal formulations containing CD73 siRNA were prepared by ethanol injection method with different N/P ratios. Following characterizing the prepared formulations (size, zeta potential, retention percentage and stability), Cy3-labeled siRNA molecules were used for efficient delivery of the siRNA molecules to 4T1 cells and toxicity was assessed by MTT assay. Real-time PCR and flow cytometry analysis were carried out to evaluate the efficacy of CD73 siRNA-containing liposomal formulations in suppressing CD73 gene expression. CD73 siRNA-containing liposomes were targeted with GE11 peptide against EGFR and its tissue biodistribution was evaluated in 4T1 breast cancer mouse model. The prepared formulation’s therapeutical efficacy for breast cancer treatment was evaluated in combination therapy with liposomal form of doxorubicin in the 4T1 breast cancer mouse model.

We have prepared and characterized an efficient cationic liposomal delivery system containing CD73 siRNA as a novel anti-tumor drug to attenuate the dual functions of CD73 in TME. The composition of cationic liposome formulation contained DOTAP, DOPE, cholesterol and mPEG2000-DSPE. All of the lipids have been extensively investigated in the literature and their biodegradability and safety parameters have been evaluated in the liposomal structures as well^35,36^. The ethanol injection method was used to encapsulate siRNA into cationic liposomes. For encapsulating hydrophilic drugs into nanolipoparticles, methods including detergent dialysis and ethanol injection are commonly applied. In our previous studies, we have successfully encapsulated siRNA against MDR1 into nanoliposomes by detergent dialysis^37^. The size of liposomes was heterogeneous and since octyl glucoside (OG) was used as the detergent of choice, this method is not considered cost-effective anymore^37^. Ethanol injection is a simple and cost-effective method which is commonly applied for liposome preparation^38^. We used this method for preparing small liposomal structures with a narrow size distribution, via injecting an ethanolic mixture of lipids into an aquatic phase containing siRNA molecules. The small size of nanoliposomes along with their near neutral surface charge, reduces the chance of opsonization and prevents uptake by the reticuloendothelial system (RES). These features increase the ability of nanoparticles to remain longer in the bloodstream. Since the size of our prepared CD73 siRNA-containing nanolipoparticles are less than 100 nm, they can easily enter solid tumors through the EPR effect. The surface charge of prepared nanoparticle’s is positive. It has been previously reported that the positive charge of cationic liposomes, ameliorates their interaction with the plasma membrane and eventually accelerates endocytosis-mediated cellular uptake^35^. However, Kim et al. have demonstrated that the transfection efficiency of cationic liposomes is mostly affected by the interactions of the liposome components with cellular membrane but not through zeta potential or size^39^. To optimize the encapsulation efficiency of our nanoliposomes, we prepared formulations with different ratios of positive to negative charges (N/P). The positive and negative charges are representative for the amine groups of DOTAP and phosphate groups of siRNA in the liposome formulation, respectively. Interestingly, we showed that by increasing the N/P ratio to 16, encapsulation efficiency can be improved to up to 94% and also the size of nanolipoparticles become smaller (85–90 nm) which is optimal for systemic administration. Our previous study on encapsulation of CpG-ODN into liposomes with N/P = 4, showed lower efficiency (below 50%) and the liposome size was more than 100 nm^31^. When the encapsulation efficiency is high, the liposome will be condensed which consequently results in smaller particle size as observed in the present study.

The results of physicochemical stability testes showed that while the mean size of liposomes containing siRNA, slightly increased after a 4-week preservation period in the refrigerator, other parameters including PDI and liposomal surface charge did not show any significant variations. Our results showed that the prepared CD73 siRNA-loaded liposomes is highly stable and that more than 50% of the loaded siRNA remained encapsulated after 24 h of incubation against PBS/FBS medium. The gradual decrease in the amount of remaining encapsulated siRNA molecules, indicates siRNA release and degradation. The stability results clearly show that the prepared liposomes containing CD73 siRNA are the optimum liposomes with long-term storage ability and physicochemical stability.

The results of uptake assay tests demonstrated efficient uptake of Cy3-labeled CD73 siRNA in both groups including liposomal Cy3-CD73 siRNA and lipofectamine—Cy3-CD73 siRNA complex (positive control). The naked form of Cy3-CD73 siRNA could not enter 4T1 cells as presented in Fig. 3, which suggests that the liposomal form of CD73 is a safe delivery system, which could efficiently deliver the siRNA molecules into 4T1 cancer cells. The knockdown efficiency of the liposomal form of CD73 siRNA is also comparable to lipofectamine complex (CD73siRNA-oligofectamine) (Fig. 4). We have also evaluated the knockdown efficiency of the liposomal form of non-targeting siRNA and Naked CD73 siRNA. The results clearly showed that none of them could significantly affect CD73 gene expression.

Cytotoxicity studies on the liposomal formulation containing 25 nM of CD73 siRNA demonstrated significant toxicity towards 4T1 cancer cells. However, the naked form of CD73 siRNA (25 nM), liposomes containing non-targeting CD73 siRNA (25 nM), and empty liposomes did not show any significant toxicity. Toxicity of CD73 siRNA-loaded liposomes suggested that CD73 downregulation could markedly decrease the proliferation of 4T1 cancer cells.

Liposomes are identified as safe drug delivery systems. Meanwhile, their safety relies on their lipid structure. It is noteworthy that previous studies have reported the toxicity of cationic liposomes containing DOTAP^39^. In the present study, in order to decrease DOTAP-induced toxicity, we used low concentrations of DOTAP (4 mM in 10 mM total lipid solution) in our prepared liposomes. As observed in our study, empty liposomes did not show any significant toxicity towards 4T1 cancer cells. It should be noted that the toxicity of cationic liposomes also depends on the studied cell line. A previous study has shown that empty and CpG-ODN-containing liposomes had no toxicity on C26 cell line, while toxic effects was observed on B16F0 cell line^31^.

Due to the importance of CD73 in tumor progression, optimizing CD73 siRNA-loaded nanoparticles could be considered a therapeutically potential strategy for treatment of solid tumors. To the best of our knowledge, this study is the first to report successful encapsulation of CD73 siRNA into cationic nanoliposomes. Niaragh et al. prepared, characterized and evaluated the effectiveness of CD73 siRNA-loaded chitosan-lactate nanoparticles in vitro and in vivo^40^. The knockdown efficiency of CD73 in their study was around 50% which is similar to our results^41^. Recently, Azambuja et al. investigated the role of CD73 in glioblastoma development by CD73 downregulation and selective CD73 enzyme inhibitor APCP (Adenosine 5′-(α, β-methylene) Diphosphate). They showed that CD73 gene silencing can more effectively suppress tumor growth in comparison to APCP^42^. It shows that CD73 is valuable anti-cancer target and that efficient CD73 gene silencing by siRNA molecules could inhibit CD73 dual functions and suggests that this approach could be a potential therapeutic strategy. CD73 and CD73-derived adenosine are involved in tumor progression, mainly through immune suppression and metastasis. Thus, we have evaluated the therapeutic efficiency of liposomal CD73 siRNA in 4T1 breast cancer mice model. In order to increase the accumulation of prepared liposomal formulations at tumor site, liposomes were targeted with GE11 peptide (via passive and active targeting) using post-insertion method and their physicochemical properties were evaluated (Table 1).

In vivo studies showed that the targeted (GE11 liposomal siRNA) and non-targeted formulations (liposomal CD73 siRNA) could efficiently suppress CD73 gene expression, which significantly reduced tumor volume and increased survival rate compared to control group. Interestingly, decreased tumor volume and increased survival rate were higher in the GE11-targeted group compared to the non-target group. Furthermore, significant reduction in tumor size and weight, observed in the isolated tumors, clearly showed the effectiveness of our liposomal formulations in inhibiting tumor growth. It has been recently reported that suppression of CD73 gene expression by siRNA, significantly reduced tumor growth in a glioblastoma tumor model, in vitro and in vivo. Azambuja et al. showed that adenosine could increase growth of glioma cancer cells via A1 adenosine receptor (A1AR)^42^. In the current in vitro study, we have also observed a significant decrease in the growth of 4T1 cancer cells following treatment with liposomal formulations compared to the control group. Azambuja et al. also reported that CD73 siRNA or CD73 inhibitor (APCP) could decrease tumor size and reduce adenosine levels up to 95% in cerebrospinal fluids (CSF), in vivo^42^. Presence of high concentrations of adenosine in hypoxic microenvironment of tumor inhibits TIL’s proliferation and function. In this regard, we observed that Lipo CD73 siRNA and GE11-Lipo CD73 siRNA did not affect the number of TILs at the tumor site compared to the control group. However, we clearly showed that downregulation of CD73, could substantially increase cytotoxic T cells (CTLs) response (significant increase in production of IFN-γ). On the other hand, CD73 downregulation efficiently decreased the Foxp3 factor of Treg cells. Similar to our findings, Jadidi et al. showed that CD73 downregulation by chitosan-lactate CD73 siRNA resulted in suppression of tumor growth, metastasis and increased survival rate. In addition, they showed that inhibition of CD73 in combination therapy with DC vaccine ameliorated secretory function of CTLs^40^.

Doxorubicin is one of the most commonly used chemotherapeutic drugs for treatment of metastatic cancers. Meanwhile, chemoresistance is a major challenge in chemotherapy of cancer patients. Overexpression of CD73 ectonucleotidase and subsequent adenosine accumulation at the tumor microenvironment is associated with TILs suppression and chemoresistance. In the present study, we also investigated the anticancer effectiveness of combinatorial treatment with the liposomal form of doxorubicin in combination with GE11 liposomal siRNA. The results showed that while Liposomal doxorubicin reduced tumor growth compared to the control group, it decreased the survival rate compared to the Lipo CD73 siRNA and GE11-Liposomal siRNA. Analysis of TILs explained the negative effect of Liposomal doxorubicin on survival rate. Injection of Liposomal doxorubicin in both groups (Liposomal doxorubicin and combination with GE11 liposomal siRNA) significantly decreased the frequency of TILs. The negative effects of chemotherapeutic agents on TILs might be considered as one of the causes of metastasis during chemotherapy which finally result in lower survival rate. Further studies are needed to elucidate the cytotoxic effect of doxorubicin on both proliferation and function TILs. Previous studies have demonstrated the relationship between chemoresistance to anthracyclines and CD73 upregulation. Loi et al. showed that increased CD73 expression is associated with doxorubicin resistance^2^. Another study on glioblastoma cells has shown that suppression of CD73 gene expression with siRNA or inhibiting its enzymatic activity by inhibitors can increase the sensitivity of tumor cells to chemotherapeutic drugs^43^. It has been shown that chemotherapy with carboplatin, doxorubicin, and paclitaxel induced CD73 and PDL1 expression. Furthermore, chemotherapy increased the ratio of Treg cells to T effector cells^14^. The results of our study also showed that downregulation of CD73 gene in both groups treated with targeted or non-targeted liposomal siRNA, significantly decreased Foxp3 levels compared to control and Liposomal doxorubicin groups. These results show that doxorubicin as a chemotherapeutic drug can play a negative and suppressive role on the immune system by increasing the proportion of Treg to T effector cells.

The negative effects of chemotherapeutic agents on TILs might be considered as one of the causes of metastasis during chemotherapy which finally result in lower survival rate. Further studies are needed to elucidate and validate the cytotoxic effect of doxorubicin on both proliferation and function TILs. Most recently Salehi Khesht et al. designed chitosan lactate nanoparticles targeted with (NPs) HIV-1 derived TAT peptide to deliver CD73 siRNA and doxorubicin to 4T1 and CT26 cancer cells. In line with our results, they showed that codelivery of CD73 siRNA with Doxorubicin potentially reduce the side effect of doxorubicin. Promote the cell death and inhibit the proliferation and metastasis of cancer cells. more importantly, accumulation of nanoparticles in TME reduced tumor growth, induced anti-tumor immune responses^44^.

## Conclusion

Taking all into account, our study showed that liposomal formulations (DOTAP, DOPE, cholesterol and mPEG2000-DSPE) with N/P = 16 have highest encapsulation efficiency. Both targeted and nontargeted formulations could significantly downregulate the CD73 gene in the in vivo studies. Moreover, targeted liposomal formulations containing CD73 siRNA alone or in combination with liposomal doxorubicin has shown potential antitumor activity in animal models. Despite recent advances in CD73 gene silencing using RNA interreference mechanisms, there is a long road ahead to translate to clinical practice. Lastly, it should be noted that our study has some limitations. To reduce the off-targeting effects of siRNA, we have used a single siRNA against CD73 in our research, which may have partly decreased the knockdown efficiency. It would be preferential to use a pool of siRNAs against the targeted genes in the preclinical studies. Furthermore, we could not use anti‐CD73 monoclonal antibody as the positive control in our in vivo studies. We suggest using a pool of liposomal siRNA molecules and compare them to the available aniti-CD73 monoclonal antibodies.

## Supplementary Information

Supplementary Information.

## References

[1] Riggio AI, Varley KE, Welm AL (2021). The lingering mysteries of metastatic recurrence in breast cancer. Br. J. Cancer.

[2] Loi S, Pommey S, Haibe-Kains B, Beavis PA, Darcy PK, Smyth MJ (2013). CD73 promotes anthracycline resistance and poor prognosis in triple negative breast cancer. Proc. Natl. Acad. Sci. U. S. A..

[3] Bandyopadhyay A, Wang L, Agyin J, Tang Y, Lin S, Yeh IT (2010). Doxorubicin in combination with a small TGFbeta inhibitor: A potential novel therapy for metastatic breast cancer in mouse models. PLoS ONE.

[4] Vijayan D, Young A, Teng MWL, Smyth MJ (2017). Targeting immunosuppressive adenosine in cancer. Nat. Rev. Cancer.

[5] Cairns RA, Harris IS, Mak TW (2011). Regulation of cancer cell metabolism. Nat. Rev. Cancer.

[6] Grzelczyk WL, Wróbel-Roztropiński A, Szemraj J, Cybula M, Pietruszewska W, Zielińska-Kaźmierska B (2019). Matrix metalloproteinases, inhibitor of metalloproteinases mRNA and protein expression in laryngeal squamous cell carcinoma. Arch. Med. Sci..

[7] Momtazi-Borojeni AA, Ebrahimi Nik M, Reza Jaafari M, Banach M, Sahebkar A (2019). Effects of immunization against PCSK9 in an experimental model of breast cancer. Arch. Med. Sci..

[8] Zara-Lopes T, Silva Galbiatti-Dias AL, Urbanin Castanhole-Nunes MM, Padovani-Júnior JA, Maniglia JV, Pavarino EC (2019). Polymorphisms in MTHFR, MTR, RFC1 and CßS genes involved in folate metabolism and thyroid cancer: A case-control study. Arch. Med. Sci..

[9] Leone RD, Emens LA (2018). Targeting adenosine for cancer immunotherapy. J. Immunother. Cancer.

[10] Khayami R, Toroghian Y, Bahreyni A, Bahrami A, Khazaei M, Ferns GA (2018). Role of adenosine signaling in the pathogenesis of head and neck cancer. J. Cell. Biochem..

[11] Heuts DP, Weissenborn MJ, Olkhov RV, Shaw AM, Gummadova J, Levy C (2012). Crystal structure of a soluble form of human CD73 with ecto-5′-nucleotidase activity. Chembiochem Eur. J. Chem. Biol..

[12] Yang J, Liao X, Yu J, Zhou P (2018). Role of CD73 in disease: Promising prognostic indicator and therapeutic target. Curr. Med. Chem..

[13] Soleimani A, Farshchi HK, Mirzavi F, Zamani P, Ghaderi A, Amini Y (2020). The therapeutic potential of targeting CD73 and CD73-derived adenosine in melanoma. Biochimie.

[14] Samanta D, Park Y, Ni X, Li H, Zahnow CA, Gabrielson E (2018). Chemotherapy induces enrichment of CD47(+)/CD73(+)/PDL1(+) immune evasive triple-negative breast cancer cells. Proc. Natl. Acad. Sci. U. S. A..

[15] Gao ZW, Wang HP, Lin F, Wang X, Long M, Zhang HZ (2017). CD73 promotes proliferation and migration of human cervical cancer cells independent of its enzyme activity. BMC Cancer.

[16] Antonioli L, Blandizzi C, Pacher P, Haskó G (2013). Immunity, inflammation and cancer: A leading role for adenosine. Nat. Rev. Cancer.

[17] Soleimani A, Taghizadeh E, Shahsavari S, Amini Y, Rashidpour H, Azadian E (2019). CD73; a key ectonucleotidase in the development of breast cancer: Recent advances and perspectives. J. Cell. Physiol..

[18] Soleimani A, Bahreyni A, Roshan MK, Soltani A, Ryzhikov M, Shafiee M (2019). Therapeutic potency of pharmacological adenosine receptors agonist/antagonist on cancer cell apoptosis in tumor microenvironment, current status, and perspectives. J. Cell. Physiol..

[19] Zuckerman JE, Davis ME (2015). Clinical experiences with systemically administered siRNA-based therapeutics in cancer. Nat. Rev. Drug Discov..

[20] Liu H-N, Qie P, Yang G, Song Y-B (2018). miR-181b inhibits chemoresistance in cisplatin-resistant H446 small cell lung cancer cells by targeting Bcl-2. Arch. Med. Sci..

[21] Joo MK, Yhee JY, Kim SH, Kim K (2014). The potential and advances in RNAi therapy: Chemical and structural modifications of siRNA molecules and use of biocompatible nanocarriers. J. Control Release.

[22] Lee SJ, Kim MJ, Kwon IC, Roberts TM (2016). Delivery strategies and potential targets for siRNA in major cancer types. Adv. Drug Deliv. Rev..

[23] Rezaee M, Oskuee RK, Nassirli H, Malaekeh-Nikouei B (2016). Progress in the development of lipopolyplexes as efficient non-viral gene delivery systems. J. Control Release.

[24] Barati, M. *et al.* Enhanced antitumor immune response in melanoma tumor model by anti-PD-1 small interference RNA encapsulated in nanoliposomes. *Cancer Gene Ther.* 1–11 (2021). 10.1038/s41417-021-00367-9 34341501

[25] Mirzavi F, Barati M, Soleimani A, Vakili-Ghartavol R, Jaafari MR, Soukhtanloo M (2021). A review on liposome-based therapeutic approaches against malignant melanoma. Int. J. Pharm..

[26] Zamani P, Navashenaq JG, Nikpoor AR, Hatamipour M, Oskuee RK, Badiee A (2019). MPL nano-liposomal vaccine containing P5 HER2/neu-derived peptide pulsed PADRE as an effective vaccine in a mice TUBO model of breast cancer. J. Control Release.

[27] Momtazi-Borojeni AA, Ebrahimi Nik M, Reza Jaafari M, Banach M, Sahebkar A (2019). Potential anti-tumor effect of a nanoliposomal antiPCSK9 vaccine in mice bearing colorectal cancer. Arch. Med. Sci..

[28] Wood H (2018). FDA approves patisiran to treat hereditary transthyretin amyloidosis. Nat. Rev. Neurol..

[29] Hay CM, Sult E, Huang Q, Mulgrew K, Fuhrmann SR, McGlinchey KA (2016). Targeting CD73 in the tumor microenvironment with MEDI9447. Oncoimmunology..

[30] Buisseret L, Pommey S, Allard B, Garaud S, Bergeron M, Cousineau I (2018). Clinical significance of CD73 in triple-negative breast cancer: Multiplex analysis of a phase III clinical trial. Ann. Oncol. Off. J. Eur. Soc. Med. Oncol..

[31] Nikoofal-Sahlabadi S, Matbou Riahi M, Sadri K, Badiee A, Nikpoor AR, Jaafari MR (2018). Liposomal CpG-ODN: An in vitro and in vivo study on macrophage subtypes responses, biodistribution and subsequent therapeutic efficacy in mice models of cancers. Eur. J. Pharmaceut. Sci. Off. J. Eur. Feder. Pharmaceut. Sci..

[32] Shahraki N, Mehrabian A, Amiri-Darban S, Moosavian SA, Jaafari MR (2021). Preparation and characterization of PEGylated liposomal Doxorubicin targeted with leptin-derived peptide and evaluation of their anti-tumor effects, in vitro and in vivo in mice bearing C26 colon carcinoma. Colloids Surf. B.

[33] Korani M, Ghaffari S, Attar H, Mashreghi M, Jaafari MR (2019). Preparation and characterization of nanoliposomal bortezomib formulations and evaluation of their anti-cancer efficacy in mice bearing C26 colon carcinoma and B16F0 melanoma. Nanomedicine.

[34] Nik ME, Malaekeh-Nikouei B, Amin M, Hatamipour M, Teymouri M, Sadeghnia HR (2019). Liposomal formulation of Galbanic acid improved therapeutic efficacy of pegylated liposomal Doxorubicin in mouse colon carcinoma. Sci. Rep..

[35] Yu B, Wang X, Zhou C, Teng L, Ren W, Yang Z (2014). Insight into mechanisms of cellular uptake of lipid nanoparticles and intracellular release of small RNAs. Pharm. Res..

[36] Zamani P, Momtazi-Borojeni AA, Nik ME, Oskuee RK, Sahebkar A (2018). Nanoliposomes as the adjuvant delivery systems in cancer immunotherapy. J. Cell. Physiol..

[37] Nourbakhsh M, Behravan J, Lage H, Abnous K, Mosaffa F, Badiee A (2015). Nanolipoparticles-mediated MDR1 siRNA delivery: Preparation, characterization and cellular uptake. Nanomed. J..

[38] Jaafar-Maalej C, Diab R, Andrieu V, Elaissari A, Fessi H (2010). Ethanol injection method for hydrophilic and lipophilic drug-loaded liposome preparation. J. Liposome Res..

[39] Kim BK, Hwang GB, Seu YB, Choi JS, Jin KS, Doh KO (2015). DOTAP/DOPE ratio and cell type determine transfection efficiency with DOTAP-liposomes. Biochim. Biophys. Acta.

[40] Jadidi-Niaragh F, Atyabi F, Rastegari A, Kheshtchin N, Arab S, Hassannia H (2017). CD73 specific siRNA loaded chitosan lactate nanoparticles potentiate the antitumor effect of a dendritic cell vaccine in 4T1 breast cancer bearing mice. J. Control Release.

[41] Jadidi-Niaragh F, Atyabi F, Rastegari A, Mollarazi E, Kiani M, Razavi A (2016). Downregulation of CD73 in 4T1 breast cancer cells through siRNA-loaded chitosan-lactate nanoparticles. Tumour Biol. J. Int. Soc. Oncodev. Biol. Med..

[42] Azambuja JH, Gelsleichter NE, Beckenkamp LR, Iser IC, Fernandes MC, Figueiro F (2018). CD73 downregulation decreases in vitro and in vivo glioblastoma growth. Mol. Neurobiol..

[43] Quezada C, Garrido W, Oyarzun C, Fernandez K, Segura R, Melo R (2013). 5′-ectonucleotidase mediates multiple-drug resistance in glioblastoma multiforme cells. J. Cell. Physiol..

[44] Salehi Khesht AM, Karpisheh V, Sahami Gilan P, Melnikova LA, Olegovna Zekiy A, Mohammadi M (2021). Blockade of CD73 using siRNA loaded chitosan lactate nanoparticles functionalized with TAT-hyaluronate enhances doxorubicin mediated cytotoxicity in cancer cells both in vitro and in vivo. Int. J. Biol. Macromol..

