# Supplementary material for: CD73 downregulation by EGFR-targeted liposomal CD73 siRNA potentiates antitumor effect of liposomal doxorubicin in 4T1 tumor-bearing mice
Source: Sci Rep. 2022 Jun 21;12:10423. doi: 10.1038/s41598-022-14392-7 (PMC9213518; doi:10.1038/s41598-022-14392-7)
Supplement: Supplementary file 1 — Supplementary Information. [file 41598_2022_14392_MOESM1_ESM.docx]

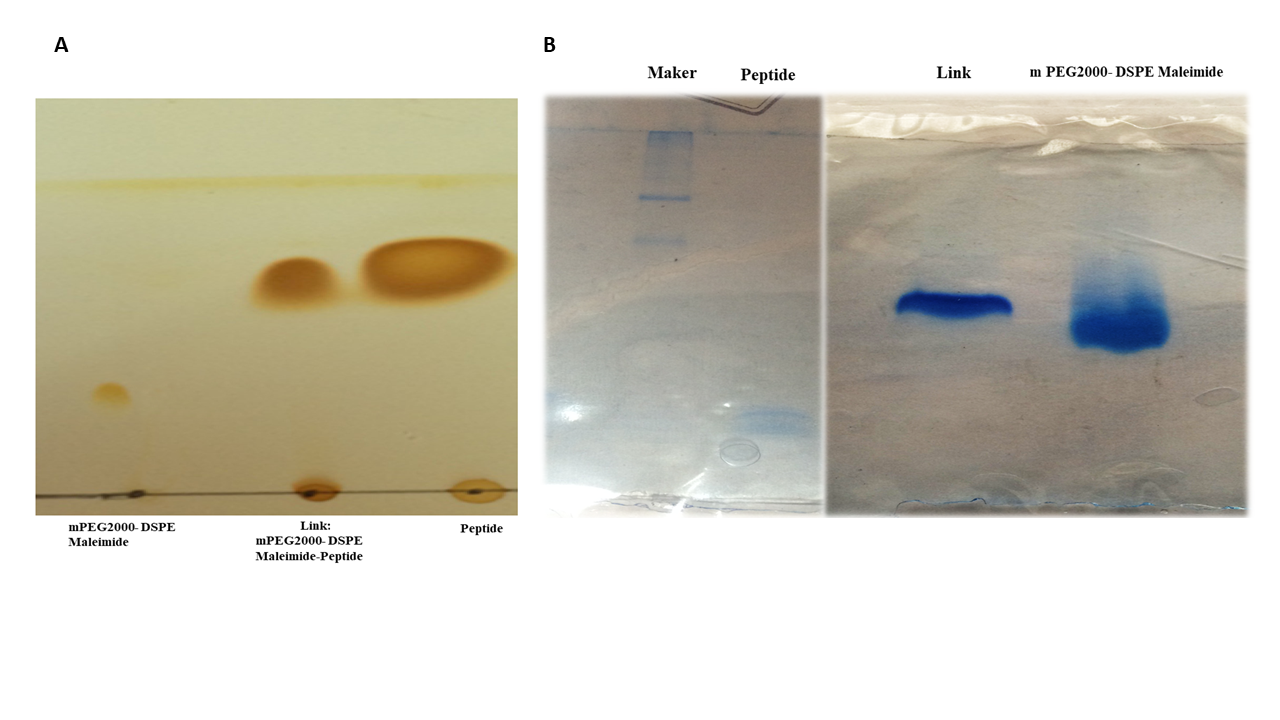


Figure1: Conjugation of GE11 peptide with mPEG2000- DSPE Maleimide. Figure 1A represents the TLC test for confirmation of linkage between GE11 and mPEG2000- DSPE Maleimide. Figure 1B also shows the confirmation of linkage by SDS PAGE test.
